# Plant-Based Inhibitors of Protein Aggregation

**DOI:** 10.3390/biom15040481

**Published:** 2025-03-25

**Authors:** Olha Zhytniakivska, Tanmay Chaturvedi, Mette Hedegaard Thomsen

**Affiliations:** 1 AAU Energy, Aalborg University, Niels Bohrs Vej 8, 6700 Esbjerg, Denmark; 2 Department of Medical Physics and Biomedical Nanotechnologies, V.N. Karazin Kharkiv National University, Svobody Sq. 4, 61022 Kharkiv, Ukraine

**Keywords:** protein misfolding, amyloid fibrils, plant-extracted compounds

## Abstract

The assembly of amyloidogenic proteins and peptides into toxic oligomeric and fibrillar aggregates is closely connected to the onset and progression of more than 50 protein diseases, such as Alzheimer’s disease, Parkinson’s disease, prion disease, and type 2 diabetes, to name only a few. Considerable research efforts at identifying the therapeutic strategies against these maladies are currently focused on preventing and inhibiting pathogenic protein aggregation by various agents. Plant-based extracts and compounds have emerged as promising sources of potential inhibitors due to their dual role as nutraceuticals as part of healthy diets and as specific pharmaceuticals when administered at higher concentrations. In recent decades, several plant extracts and plant-extracted compounds have shown potential to modulate protein aggregation. An ever-growing body of research on plant-based amyloid inhibitors requires a detail analysis of existing data to identify potential knowledge gaps. This review summarizes the recent progress in amyloid inhibition using 17 flavonoids, 11 polyphenolic non-flavonoid compounds, 23 non-phenolic inhibitors, and 59 plant extracts, with the main emphasis on directly modulating the fibrillation of four amyloid proteins, namely amyloid-β peptide, microtubule-associated protein tau, α-synuclein, and human islet amyloid polypeptide.

## 1. Introduction

During the last few decades, misfolding protein disorders have become global epidemics affecting the quality of life of millions worldwide, with profound social and economic outcomes and no effective therapy so far. These include the broad spectrum of neuropathic, metabolic, or aging-related disorders such as Alzheimer’s, Parkinson’s, Creutzfeldt–Jacob’s, Huntington’s, prion diseases, amyotrophic lateral sclerosis, and type II diabetes, to name only a few [1,2,3]. Although the precise etiology of amyloid-associated maladies is different and is still not fully understood owing to its complicity, recent advances have demonstrated that the deposition of the insoluble proteinaceous plaques, amyloid fibrils, is the common pathological hallmark of protein misfolding disorders [4,5,6,7,8,9,10,11]. More specifically, Alzheimer’s disease is marked by the presence of extracellular senile plaques composed of the fibrillar amyloid-β peptide (Aβ) and intracellular neurofibrillary tangles composed of microtubule-associated protein tau [4,5,6,7]; the aggregated α-synuclein is the major component of Lewy bodies observed in Parkinson’s diseases; type II diabetes is characterized by the aggregation of amylin in the islets of Langerhans [10,11], etc. Despite the different structures of disease-associated amyloid proteins, the fibrillar assemblies share a typical cross beta-sheet architecture, in which individual beta-strands run perpendicular to the long fibril axis [3,4,5,12,13]. According to the amyloid cascade hypothesis proposed by Hardy and Higgins in 1992 to explain the pathogenesis of Alzheimer’s diseases, the accumulation and deposition of the amyloid-beta protein in the brain serve as the primary trigger for neurodegeneration and cognitive decline [14]. Later, Lesné et al. demonstrated that memory deficits in the mouse model of AD appear before detectable plaque formation, suggesting that soluble oligomeric forms of Aβ are more toxic than mature amyloid fibrils [15]. Currently, the revised amyloid cascade hypothesis, which identifies protein oligomers as the primary drivers of neurotoxicity in numerous neurodegenerative diseases, is more widely accepted [16,17,18,19,20]. These oligomers contribute to plasma and intracellular membrane disruption, promote the formation of reactive oxygen species, and induce mitochondrial dysfunction [16,17,18,19,20].

To the best of our knowledge, there are no effective therapeutic strategies against protein-misfolding diseases. Up to now, the treatment of amyloidosis has provided only short-term symptomatic relief. However, accumulating evidence indicates that the inhibition of protein aggregation is a relevant target in developing treatments against protein-misfolding disorders [21,22,23]. Considerable research efforts are currently focused on assessing the possibility of preventing pathological protein aggregation and reducing the amyloid-related cytotoxicity by various agents, namely nanoparticles [24,25,26,27], peptides [28,29], fluorescent dyes [30,31,32,33], antibodies [34], and natural compounds [35,36,37,38,39,40,41]. Among the above, the plant-derived natural extracts and plant-extracted compounds are the most prospective due to their favorable properties such as (i) lower cytotoxicity in comparison with the majority of synthetic compounds; (ii) availability at affordable prices; (iii) they can be consumed as part of a healthy diet to prevent amyloid-associated diseases; and (vi) they are multifunctional, possessing anti-amyloid, anti-inflammatory, and antioxidant properties simultaneously [42,43,44].

An ever-growing body of research on plant-based amyloid inhibitors requires a detail analysis of existing data to identify potential knowledge gaps in the field, enabling the development of more effective therapies and strategies. The present review outlines recent advances in plant-based formulation potential to inhibit amyloid fibril formation of four amyloid proteins, namely the amyloid-β peptide, microtubule-associated protein tau, α-synuclein, and human islet amyloid polypeptide. More specifically, using the available public databases, we selected and discussed the inhibition mechanism and the anti-amyloid effectiveness of 17 flavonoids, 11 polyphenolic non-flavonoid compounds, 23 non-phenolic inhibitors, and 59 plant extracts, with the main emphasis on the direct modulation of protein fibrillation.

## 2. Molecular Mechanism of Amyloid Fibril Inhibition

Although, the precise mechanism of amyloid fibril formation is not completely clear, numerous studies indicate that the Aβ peptide, tau protein, synuclein, and human islet amyloid polypeptide self-assemble through similar pathways, and the cascade of their aggregation consists of three consecutive steps: (i) initial nucleation, which corresponds to the transition of monomeric proteins to amyloidogenic nuclei; (ii) the elongation phase, which consists of the formation of protofibrils; and (iii) the saturation phase, which is characterized by the assembling of protofibrils to mature amyloid fibrils [3,5,45,46,47,48]. The aggregation of Aβ, tau, α-Syn, and IAPP also includes the process of secondary nucleation with the fragmentation of protofibrils to short fragments [49,50,51,52,53]. Secondary nucleation is believed to be an essential factor that affects the kinetics of amyloid formation since fragments seed further fibril growth and initiate an autocatalytic replication of the amyloid structure [49,50,51,52,53]. This alternative “seed” pathway of fibril formation is more favorable and less energy-demanding in comparison with monomer–monomer nucleation. It is believed that secondary nucleation is crucial for spreading amyloid pathology between cells and tissues [54], especially in the case of tau proteins, which can spread between cells and accelerate the protein aggregation process in neighboring cells [55].

**Figure 1:**
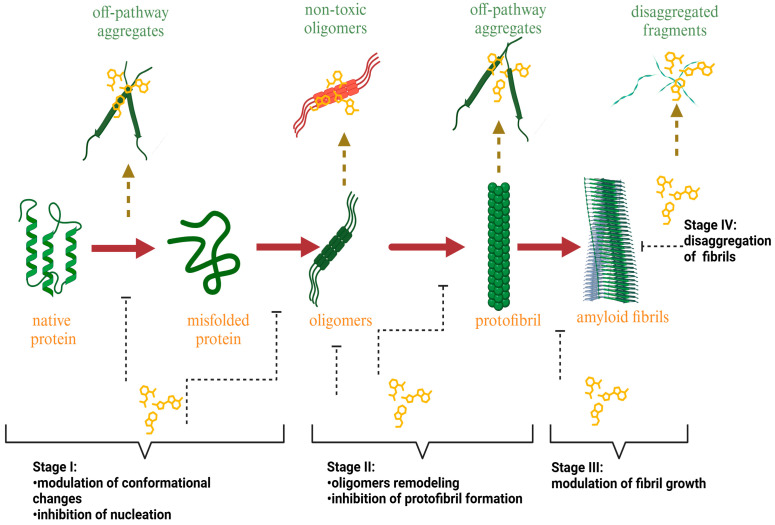
Plant-extracted compounds can interfere with the protein self-assembly process and reduce amyloid fibrils formation during the different steps of the aggregation cascade. Created in BioRender at https://app.biorender.com/ (accessed on 6 March 2025).

Although the biomolecular details of plant compound suppression and modulation of amyloid fibrils as well as the direct amyloid species involved in the aggregation process are not fully understood, numerous studies indicate that plant bioactive compounds can inhibit or diminish the fibrillation process in all stages of protein aggregation (Figure 1).

1. ***The suppression of the early stages of fibril formation* via *the modulation of protein gathering and nuclei formation***: In this pathway, the plant-derived inhibitor can alter fibril formation in at least two ways: (i) by increasing the protein’s stability in the monomeric form and (ii) by complexation with the monomer, thereby preventing monomer–monomer interactions and the conversion of α-helical protein intermediates to the β-sheet structures [56,57,58,59,60,61,62,63,64]. To exemplify, Wang et al. found that the major flavonoid of Scuttellaria baincalensis Georgi baicalein and baicalin inhibit IAPP fibrillation through concentration-dependent suppression of protein nucleation, thereby preventing the formation of β-sheet-rich aggregates [56]. Myricetin prevents the conformational change in the Aβ protein from a random coil to a β-sheet-rich structure by preferentially targeting Aβ monomers [57]. It was hypothesized that the ability of epigallocatechin gallate to inhibit the formation of the IAPP amyloid fibrils relates to the suppression of the early stages of protein self-aggregation, presumably IAPP dimer formation [59].
2. ***The stabilization of protein oligomerization and the inhibition of pre-fibril formation*:** Plant bioactive compounds can suppress the formation of prefibrillar structures by stabilizing protein oligomers [65,66,67,68,69]. Moreover, recent studies indicate that natural amyloid inhibitors can remodel soluble protein oligomers into non-toxic protein aggregates [70,71,72,73,74,75]. Brazilin, a natural compound extracted from *Caesalpinia sappan*, serves as a typical example of amyloid inhibitors capable of suppressing the formation of the toxic-on pathway of Aβ oligomers, remodeling them to β-sheet aggregates with a higher molecular weight (above 70 kDa) [70]. Myricetin, tannic acid, and nordihydroguaiaretic acid also demonstrated an ability to remodel the amyloid fibrils, neutralizing the oligomer-specific conformational epitope at substoichiometric concentrations [71].
3. ***The stabilization of protofibrils***: Several natural compounds can stabilize the pre-fibrillar state, thus blocking further fibril growth [76,77,78,79]. Quercetin was shown to inhibit the fibrillization of the IAPP, slowing down the growth of the amyloid fibrils after 10.5 h of incubation and causing an increase in the fibril amount wherein the fibril size remained constant [76]. Moreover, nordihydroguaiaretic acid inhibits the direct protofibril–protofibril association of Aβ but does not alter protofibril elongation through monomer addition [78].
4. ***The disassembly or remodeling of the mature amyloid fibrils***: Many compounds were shown to inhibit the formation of amyloid fibrils, destabilizing the preformed protein assemblies by subsequently converting the mature fibrillar structures to non-toxic smaller intermediates [72,80,81,82,83,84,85,86]. For example, the ThT assay and electron microscopy studies demonstrated that baicalein disaggregates preformed mature amyloid fibrils of the Aβ peptide to an amorphous state [80]. Palhano and colleagues observed that epigallocatechin-3-gallate (EGCG) remodels mature amyloid fibrils of Aβ_1–40_ and IAPP_8–24_ through the formation of a Schiff base and hydrophobic interactions [81].

In an attempt to understand the precise mechanism of amyloid inhibition through plant bioactive compounds, numerous studies in the past decades were directed to the investigation of the molecular interactions involved in the complexation between protein and anti-amyloid agents. Although the type and specificity of the interactions depend on the plant-extracted compound, the protein structure, and the stage of aggregation that was targeted [87,88,89,90,91,92,93,94,95,96], both covalent or noncovalent (hydrophobic interactions, hydrogen bonding, π-π interactions, and charge–charge interactions) interactions were observed in protein aggregation inhibition by bioactive compounds [97,98,99,100,101]. To exemplify, some flavonoids and catechol-containing compounds form covalent o-quinone-mediated adducts with amyloidogenic proteins that lead to the blocking of protein aggregation or the dissolution of preformed amyloid fibrils [71,87,88,89,90,91,92,93,94]. The non-catechol-derived covalent mechanisms between the nucleophilic thiols and amines of amyloid-forming proteins and electrophilic-reactive groups of inhibitors were also observed [95,96]. Li et al. hypothesized that the formation of the covalent adduct via a Schiff base between the aldehyde-reactive functional group in oleocanthal and the lysine residue of the hexapeptide VQIVYK sequence in tau proteins could be responsible for the oleocanthal inhibition of tau aggregation [95]. Cinnamaldehyde prevents tau fibrillation through the covalent mechanism via the involvement of adduct formation between the unsaturated carbon on cinnamaldehyde and cysteine thiols of tau [96]. Numerous studies provided support to the involvement of both covalent and non-covalent interactions in protein inhibition by naturally occurring compounds [81,95,96,97,98,99]. The non-covalent interaction involved in inhibiting protein fibrilization by plant active compounds is highly specific and varies significantly for different plant compounds and protein species [97,98,99]. We summarized the inhibition potential and the mechanism of inhibition for the most promising and extensively studied plant-derived molecules.

## 3. Polyphenols as Amyloid Inhibitors

### 3.1. Flavonoids

Flavonoids, found in plant food and several beverages, such as tea and wine, are the most abundant polyphenol antioxidants in human diets, possessing a well-known wide spectrum of beneficial activities, including anti-cancerogenic, anti-inflammatory, antidiabetic, and antioxidant, to name only a few. Recent studies demonstrated the potential of several naturally occurring flavonoids in amyloid inhibition studies against many amyloidogenic proteins [80,100,101,102,103,104,105,106,107,108,109,110,111,112,113,114,115,116,117,118,119,120,121,122,123,124,125,126,127,128,129,130,131,132,133,134,135,136,137,138,139,140,141,142,143,144,145,146,147,148,149,150,151,152,153,154,155,156,157,158,159,160,161,162,163,164,165,166,167,168,169,170,171,172,173,174,175,176,177,178,179,180,181]. Though the biomolecular mode of flavonoid action in protein aggregation remains elusive, numerous investigations substantiated the destabilization and diminishing of protein fibrillization in the presence of flavonoids [80,100,101,102,103,104,105,106,107,108,109,110,111,112,113,114,115,116,117,118,119,120,121,122,123,124,125,126,127,128,129,130,131,132,133,134,135,136,137,138,139,140,141,142,143,144,145,146,147,148,149,150,151,152,153,154,155,156,157,158,159,160,161,162,163,164,165,166,167,168,169,170,171,172,173,174,175,176,177,178,179,180,181]. The amyloid inhibition potential and the possible mechanism of protein aggregation suppression for the effective flavonoids are presented in Table 1. An overview of some important and intensively studied flavonoids is presented below in Table 1.

The neuroprotective effect of flavonoid catechins, observed in high amounts in green tea, has been widely studied [100,101,102,103,104,105,106,107,108,109,110]. The most abundant representatives such as (–)-epicatechin (EC), (–)-epigallocatechin (EGC), (–)-epicatechin gallate (ECG), and (–)-epigallocatechin gallate (EGCG) demonstrated effective amyloid inhibition potential [100,101,102,103,104,105,106,107,108,109,110]. The collective evidence from recent studies has revealed that **epigallocatechin gallate (EGCG**) possesses the highest neuroprotective properties from the catechins [101,102]. EGCG effectively inhibits the amyloid formation of several amyloidogenic proteins by preventing the aggregation of α-Syn, Aβ, IAPP, and tau in the early stages of protein aggregation [58,81,100,101,102,103,104,105,106,107,108,109,110,111,112,113,114,115]. The flavonoid also remodels toxic prefibrillar oligomers and mature amyloid fibrils to the non-toxic amorphous species [58,81,100,101,102,103,104,105,106,107,108,109,110,111,112,113,114,115]. Ehrnhoefer et al. have concluded that EGCG binds to intrinsically disordered proteins (α-syn and Aβ) most probably via nonspecific backbone interactions, preventing the conversion of random-coil structures into β-sheets [103]. The potential of EGCG to change the aggregation pathway of α-synuclein into the formation of non-toxic protein aggregates was observed by Bieschke et al. [104]. Cao and Raleigh reported that EGCG inhibits the amyloid formation of IAPP in the initial stage of IAPP nucleation and remodels the mature fibrils, presumably through hydrogen bonding to the peptide backbone and through relatively nonspecific, hydrophobic interactions with side chains [58]. Wobst et al. demonstrated the ability of EGCG to prevent the aggregation of tau into toxic oligomers at ten- to hundred-fold substoichiometric concentrations [105]. CryoEM studies performed by Seidler et al. pointed to the EGCG-induced disaggregation of tau fibrils by EGCG wedging into a cleft at the interface of two protofilaments of the paired helical filament, thus causing charge repulsions between tau layers of the fibrils [106]. The dual effect of EGCG on aggregation inhibition and full-length tau protein fibril disassembly was also demonstrated by Sonavane et al. [107].

Despite the structural differences between the Aβ peptide, tau, α-Syn, and IAPP, numerous studies pointed to the primary role of non-covalent interactions (presumably hydrogen bonds and hydrophobic interactions) in EGCG-protein inhibition [108,109,110,111]. Specifically, the molecular dynamics simulation studies between EGCG and the pentamer or decamer of α-Syn indicate the EGCG-induced remodeling of the α-Syn fibrils, presumably through hydrophobic interactions and hydrogen bonding with three different fibril sites involving the residues K58, E61, T64, K96, and D98 [108]. Yao et al. demonstrated that EGCG reduces the overall β-sheet content and disrupts the structure of the α-Syn fibrils that target β-sheet region 1 (residues 45–55) and region 3 (residues 61–95), which are important for protein aggregation [109]. More specifically, it was observed that the disruption of α-Syn fibrils occurred through (i) hydrogen bonding interactions with the charged synuclein residues E46, E61, K80, and E83 and polar residue S87; (ii) strong hydrophobic interaction between EGCG and the V66 residue; (iii) H-bond formation of EGCG with E46 and K80 inducing the disturbing of the E46–K80 salt bridge, thus reducing fibril stability; and (iv) the formation of a stacking pattern between ring B, ring GA, and the indazole ring of H50 of the protein. [109]. Nonspecific interactions are also assumed as the main contributors in the process of tau–EGCG inhibition [106]. It was observed that EGCG formed polar and hydrogen-bond contacts with residues N327, H329, E338, and K340 located in the polar clef at the intersection of the two protofilaments of tau fibrils [106]. The EGCG–tau interaction is additionally stabilized by π-π interactions of the stacked aromatic rings of EGCG [106]. The major role of hydrogen bonding and hydrophobic interactions in the EGCG–tau complexation was also confirmed by molecular modeling studies performed by Sonawane et al. [107]. The ability of EGCG to inhibit the early stages of IAPP fibrillization was driven by hydrogen bonds, Pi-alkyl, Van der Waals forces, and carbon–hydrogen bonds between EGCG and the residues R11, L12, S19, A25, and I26 that are localized between the coil and helix of the hIAPP protein [110]. EGCG undergoes nonspecific hydrophobic and hydrogen bonding interactions with Aβ peptide that can mediate its anti-amyloid activity [111,112,113,114,115]. Liu et al. showed that hydrogen bonds presumably occur between EGCG and the protein backbone, whereas the hydrophobic interactions were observed in the protein side chains [111]. In addition, it was hypothesized that the ability of EGCG to prevent the conformation of Aβ α-helices into β-sheets is also driven by the van der Waals interactions with amino acid residues F4, R5, F19, F20, E22, K28, G29, L34, M35, V36, G37, and I41 [111]. In addition, the extensive atomistic replica exchange molecular dynamics simulations of Aβ1-42 dimer in the presence and absence of EGCG performed by Zhang et al. demonstrated the strong intermolecular interactions with residues F4, F19, F20, T10, I31, I32, M35, V36, V39, and I41 on the side chains as well as the number of contacts between three aromatic rings of EGCG and residues G29, A30, G37, G38, V39, and A42 on the backbone [112]. Moreover, the hydrophobic interactions were assumed to play the primary role in the EGCG-induced remodeling of mature amyloid fibrils [81]. The remodeling process also involves Schiff base formation between EGCG and amyloid fibrils through a reaction with free amines in the protein [81]. Moreover, EGCG-induced interference in the central region of Aβ (F19 and L34) leading to the break of the hydrogen bond between the H6 and E11 residues and the salt bridge with the side chain of K28 is crucial for the disruption of the amyloid structure [113,114,115].

**Baicalein** is another representative of flavonoids demonstrating effective modulation of the amyloid assembly in various studies [80,117,118,119,120,121,122,123,124]. More specifically, it has the ability (i) to inhibit hIAPP fibrilization through concentration-dependent suppression of nucleation, thereby preventing the formation of oligomeric species and β-rich fibrils [123,124]; (ii) to modulate tau aggregation by inducing off-pathway oligomers [90]; (iii) to suppress α-Syn fibrillation and protect SH-SY5Y cells from α-Syn-induced cytotoxicity [80,89,119,120,122]; (iv) to affect Aβ oligomerization and fibrillation and prevent amyloid-induced toxicity in PC12 cells [80,121]; and (v) to disaggregate the existing fibrils in a dose-dependent manner [80,89,90,117,118,119,120,121,122,123,124]. Multiple studies’ insights indicate that the baicalein inhibition effect is primarily attributed to the formation of the baicalein-protein covalent adducts via a Schiff base mechanism [80,89,90,91,118,119,120,121,122,123,124]. Specifically, Zhu et al. demonstrated that the complexation of baicalein with α-synuclein led to the formation of the soluble oligomers of α-synuclein, in which the protein molecules have been covalently modified by baicalein quinone, thus forming a Schiff base with a lysine side chain in α-synuclein [89]. MALDI-TOF analysis performed by Sonawane et al. pointed to tau inhibition through covalent modification with the possible involvement of the adjacent hydroxyl groups of baicalein [90]. Similar conjugation reactions were reported in the case of amylin and Aβ-peptide aggregation inhibition by baicalein [80,121,122,123,124]. The anti-amyloid activities of baicalein are significantly reduced under anaerobic conditions and enhanced when it is autooxidized into the quinone form of baicalein [89]. Screening a library of natural polyphenolic compounds uncovered a significant role of the vicinal hydroxyl groups in the baicalein inhibition of amylin [123]. Though multiple studies indicate the covalent baicalein-protein interactions as a driving force of baicalein inhibition of aggregation and disaggregation of amyloid proteins, they do not rule out potential noncovalent interactions as additional mechanisms. Yao et al. demonstrated that baicalein destructs α-synuclein fibrillar aggregates by disrupting the E46-K80 salt bridge and β-sheets in the N-terminal regions (residues 39–51) and C-terminal regions (residues 91–96) and by remodeling the inter-protofilament interface [120]. More specifically, the authors concluded that the fibril disruption by baicalein was driven by the aromatic stacking interactions with residues Y39 and F94, strong hydrophobic interactions with the V40/V66/V82 residues, and H-bonding interactions with E46 [120]. The molecular dynamics simulation demonstrated that baicalein binds to the hexapeptide repeat region (244–373) of the tau protein primarily through hydrophobic interactions with subsequent stabilization of baicalein-protein complexes via the water-mediated hydrogen bond [90]. Notably, the disruptive effects of baicalein of α-synuclein fibrils are polymorphism-dependent [120]. Specifically, Yao et al. observed that baicalein destroys wild-type α-synuclein fibrils by disrupting the E46-K80 salt bridge and remodeling the inter-protofilament interface [120]. In contrast, the disruption of two familial PD-associated mutants’ (E46K and H50Q) α-Syn fibrils is initiated at the E61-K80 salt bridge and N-terminal β-sheet (E46K) or the inter-protofilament interface and N-terminal β-sheet (H50Q) [120].

**Quercetin**, a readily available naturally occurring flavonoid, possesses the same core nucleus as baicalein but differs in the number and positioning of OH moieties. Quercetin has been previously shown to inhibit the amyloid formation of Aβ, α-synuclein, and IAPP proteins; disaggregate the performed amyloid fibrils; and reduce Aβ(1–42)-induced toxicity in PC12 cells [61,76,82,94,125,126,127,128,129,130]. More specifically, in vitro studies revealed that quercetin suppresses the aggregation of Aβ and disrupts the mature fibrils by forming hydrophobic interactions and hydrogen bonds with the β-sheet structure of the protein [126]. Notably, quercetin binds towards the middle of the Aβ protein monomer, forming hydrogen bonding primarily within the B-ring hydroxyl groups and in proximity to the key Lys 16 residue [130]. Another study suggests that quercetin binds β-amyloid oligomers at the early stages of their aggregation, which leads to the formation of modified oligomers and hinders the creation of β-sheet structures [41]. The occurrence of favorable interactions between quercetin and Aβ40 at the first steps of the nucleation was also observed by Alvarez-Berbel et al. [61]. More specifically, quercetin reduced the elongation rate of the normal fibrillation pathway and lead to the generation of off-pathway Aβ-quercetin oligomers [61]. Zhu et al. demonstrated that quercetin suppresses the fibrillation of α-synuclein and disaggregates preformed fibrils by inhibiting the fibril growth step [127]. The possible mechanism of α-synuclein inhibition involves the covalent binding of quercetin with the protein, leading to the formation of the covalently modified α-synuclein oligomers and aggregates with increased hydrophilicity and higher stability [127]. It was found that pre-incubated quercetin exhibited a noticeably stronger inhibition behavior to fibril formation than that of the freshly prepared sample. The comparative analysis of the α-synuclein inhibition kinetics by the ortho-, meta-, and para-isomer of quercetin reveals the importance of the ortho-dihydroxyl group for fibril inhibition [127]. In addition, quercetin inserts the inhibitory effects on IAPP fibrillization, presumably affecting the lag phase of IAPP aggregation by stabilizing the monomeric protein and reducing the unfolding and subsequent nucleation of IAPP [76]. Furthermore, quercetin shifts the thermodynamic equilibrium of mature fibrils toward the reformation of monomeric IAPP and disaggregates preformed IAPP fibrils through the formation of phenolic/IAPP monomer complexes [76]. Nonpolar interactions play an important role in the quercetin–IAPP complexation. Abioye et al. demonstrated that quercetin binding occurs in the hydrophilic pocket of the protein and is facilitated by a network of four hydrogen bonds involving R-11 and N-21 of the IAPP monomer [76]. The molecular docking and molecular dynamics simulation study of quercetin–IAPP interactions indicate that flavonoids can disaggregate chain A and chain B of hIAPP by binding with two regions of hIAPP, L12–A13–N14 and N31–V32–G33–S34–N35 [82]. King et al. utilized MD simulations to probe the ability of quercetin to inhibit the aggregation of IAPP(20–29) monomers into a trimer [131]. They observed that flavonoid inhibited interpeptide F23 interactions principally through π interactions, indicating a key role of aromaticity in assessing inhibition efficacy [131].

**Myricetin**, one of the most studied polyphenols, has been found to possess antioxidant, anti-inflammatory, anti-tumor, anti-dementia, and neuroprotective effects [132,133,134]. Numerous studies confirmed the ability of myricetin to interfere with amyloid protein aggregation and toxicity [66,71,76,94,132,133,134,135,136,137,138,139,140,141,142,143,144]. More specifically, myricetin has been shown to dose-dependently inhibit Aβ40 and Aβ42 oligomerization by interacting specifically with protein monomers [66]. Moreover, myricetin stabilizes the Aβ monomer’s transition from the statistical coil to the β-sheet by binding to the monomeric Aβ peptide region containing S8, G9, H3, K16, D23, and I31 residues [66]. Remarkably, Ladiwala et al. demonstrated that myricetin rather remodels prefibrillar oligomers and fibrils into unstructured, insoluble aggregates than prevent the aggregation of Aβ [71]. The authors concluded that myricetin promoted nonspecific interactions between Aβ monomers and remodeled existing soluble oligomers into SDS-stable disordered aggregates [71]. Recent studies on transgenic mice demonstrated that a prolonged administration of myricetin increased the levels of soluble Aβ monomers and significantly decreased the amount of fibrillar deposits [136]. Myricetin was found, and it also serves as an effective inhibitor of tau fibrillization [138,140]. In vitro studies indicates that a flavonoid compound blocks tau aggregation by interfering with the elongation phase of protein assembly [140]. Moreover, myricetin slows liquid–liquid phase separation of full-length tau proteins and significantly inhibits subsequent tau aggregation [138]. The molecular dynamics study demonstrated that myricetin’s ability to interfere with tau aggregation could be connected with the flavonoid-induced conformational changes in the oligomer aggregate, leading to the disruption of amyloid H bonding [142]. Myricetin also inhibits the formation of α-synuclein oligomers and fibrils in a dose-dependent manner and disaggregates preformed protein aggregates [143]. Insights from photo-induced cross-linking of unmodified proteins, circular dichroism spectroscopy, the electron microscope, and the atomic force microscope studies delineate that myricetin inhibited α-syn oligomerization and secondary structure conversion by binding to the N-terminal region of the protein [143]. The inhibition of α-synuclein oligomerization is believed to be an important pathway in myricetin-induced amelioration of α-synuclein synaptic toxicity [143]. Myricetin also demonstrated the potential to inhibit hIAPP fibrillization and protect mammalian PC12 cells from protein-induced cytotoxicity [144]. Molecular modeling studies indicate that myricetin, which is similar to quercetin, inhibited interpeptide F23 interactions principally through π interactions, indicating a crucial role of aromaticity in assessing inhibition efficacy [131].

Accumulating evidence demonstrated the effectiveness of **taxifolin** as a potential agent in the prevention and treatment of amyloid-associated diseases and as a modulator of amyloid assembly in various in vitro or in vivo studies [94,131,155,156,157,158]. More specifically, Sato et al. observed that the catechol-type flavonoid taxifolin suppresses the elongation phase of Aβ42 aggregation through a site-specific mechanism, in which a catechol structure could be initially autoxidized to form the o-quinone on the B-ring, followed by the formation of the o-quinone–Aβ42 adduct that targets Lys residues at positions 16 and 28 of Aβ42 [94]. Moreover, the formation of the Aβ42–taxifolin adduct also contributes to the destabilization of Aβ42 fibrils [94]. By disclosing the molecular and structural basis responsible for Aβ42–taxifolin adduct formation, the molecular modeling study by Ginex et al. supported the binding of (+)-taxifolin to the hydrophobic groove delimited by the edges defined by the L16 and Q22 residues in the fibril [157]. More specifically, they obtained that the aza-Michael addition of the o-quinone species of (+)-taxifolin with Aβ42 fibrils proceeds through the nucleophilic attack of the deprotonated amino group of a K16 residue, as well as by a water-assisted proton transfer, which is the rate-limiting step of the reaction [157]. In addition, recent studies demonstrated that the application of taxifolin could mitigate the accumulation of amyloid fibrils and improve memory capacity in vivo [158]. To the best of our knowledge, the potential of taxifolin to suppress the amyloid formation of synuclein and tau of IAPP is purely investigated. Likewise, molecular dynamics simulations of the ability of dihydroquercetin to probe the aggregation of IAPP trimer reveal the reduction in β-strand formation through interactions between the carbonyl oxygen of flavonoids and the 23 residues in the IAPP [131].

Numerous studies suggest predominant structural requirements that appear necessary to provide a flavonoid the ability to inhibit the fibrillation of the amyloidogenic proteins. Recently, some research groups have reported that flavonoids with catechol moieties such as (+)-taxifolin, myricetin, quercetin, baicalein, and epigallocatechin gallate were more active inhibitors of protein fibrillation compared to the non-catechol-containing compounds [76,92,94,130,135,136,140,165,181]. Based on the previous reports, the higher amyloid suppression potential of catechols was connected with their site-specific inhibition mechanism requiring the initial autooxidation of the catechol moiety into o-quinone with subsequent covalent o-quinone–Aβ-42 adduct formation targeting lysine residues at positions 16 and 28 [66,92,94,100,127,136]. Moreover, the hydroxylation in the B-ring of flavonoids (at the 3′, 4′, and 5′ positions) appeared crucial for their amyloid anti-aggregation efficacy [94,126,130]. More specifically, Marsh et al.’s comparative analysis of the inhibition activity of a discreet set of compounds pointed out that the hydroxylation in the B-ring is the most important determinant of their action against Aβ amyloid [130]. Additional confirmation of the assumption that restricting orthoquinone formation to the B-ring can increase the specificity of flavones for Aβ peptide comes from the work of Sato et al. [94]. The comparative analysis of quercetin and apigenin’s ability to inhibit Aβ40 aggregation reveals the higher inhibition potential of quercetin in comparison with apigenin, which was connected with the presence of more hydroxyl groups capable of forming hydrogen bonds with the peptide backbone [131]. The structure–activity analysis of the IAPP aggregation inhibition of the major bioactive compounds of Scutellaria baicalensis Georgi demonstrated the higher IAPP anti-amyloid effect of baicalein in comparison with wogonin and oroxylin A, indicating that the flavonoids with the ortho-hydroxyl group on the B-ring are also more effective against hIAPP nucleation than those without this structural feature [56].

### 3.2. Non-Flavonoid Polyphenolic Compounds

Many of the non-flavonoid phenolic compounds identified from our search exhibit anti-amyloid effects [65,67,72,182,183,184,185,186,187,188,189,190,191,192,193,194,195,196,197,198,199,200,201,202,203,204,205,206,207,208,209,210,211,212,213,214,215,216,217,218,219,220,221,222,223,224,225,226,227,228,229,230,231,232,233,234,235,236,237,238,239,240,241,242,243,244]. In this review, we highlight some non-flavonoid phenolic natural compounds which have been reported in diverse model protein systems to have the potential to inhibit the aggregation of amyloidogenic proteins (Table 2).

**Curcumin**, a polyphenolic natural compound derived from the *Curcuma longa* plant, has a therapeutic potential towards amyloid-connected disorders, preventing amyloid formation and amyloid-induced cytotoxicity [35,36,182,183,184,185,186,187,188,189,190,191,192,193]. Several studies have documented the ability of curcumin to mitigate AD pathology by (i) suppressing Aβ aggregation, presumably by blocking the formation of protein oligomers through binding to the N-terminus of Aβ monomers [184,185,186]; (ii) disrupting fibrillar aggregation in vitro or in vivo [184,185,187,188]; (iii) forming non-toxic “off-pathway” soluble oligomers and prefibrillar aggregates of Aβ [36,184]; (iv) blocking the toxicity of Aβ oligomers in SH-SY5Y neuroblastoma cells [184]; and reducing toxicities induced by Aβ species [187]. Moreover, curcumin was demonstrated to cross the blood–brain barrier, thereby labeling the senile plaques in the AD brain [184,188]. Numerous studies pointed to the non-covalent curcumin–Aβ interactions as the main driving force of protein inhibition and disaggregation [189,190,191,192,193]. The all-atom explicit solvent molecular dynamics simulation study reveals that curcumin decreases the antiparallel beta-sheet structure of Aβ oligomers but does not influence the monomer–monomer contacts of monomers [189]. It was demonstrated that the curcumin-induced reduction in the beta-sheet structure of Aβ oligomers is associated with its penetration to the hydrophobic core of the Aβ dimer, thereby causing the perturbation and deformation of β-sheet secondary structures, presumably by the pi-stacking interactions between the curcumin keto or enol ring with the protein aromatic residues (Tyr, Phe, and His) and the amide-carbonyl hydrogen bonds (Val24, Asn27, Glu11, Gln 15, Leu34, and Ile41) [189]. According to the previous findings, the KLVFFA motif in the Aβ structure is the essential binding site for curcumin [190,191]. Solid-state NMR analysis indicates that curcumin breaks the β-sheet structure interacting with the KLVFFA sequence pattern via hydrophobic forces and hydrogen bonding [190,191]. Similar results were obtained by Rao et al. using molecular docking and molecular dynamics studies between curcumin and (i) amyloidogenic steric zipper Aβ hexapeptides and (ii) full-length Aβ fibrils [192]. More specifically, curcumin forms inter-residue side chain interactions with the segment KLVFFA of the hexapeptide octamer fragment of Aβ and the residue HQKLVFFA in full-length amyloid-β peptide via hydrophobic interactions with the spine of the Ab fibril interface, hydrogen bonding between the 3-hydroxy and 4-methoxy substituents and polar lysine amino acid side chains, and π–π stacking interactions between the imidazole side chain of histidine (^14^H) and aromatic rings of curcumin [192].

Curcumin can ameliorate AD pathology by disrupting and inhibiting the tau tangles [83,193,194]. Rane et al. demonstrated that curcumin inhibits tau fibrillization in the initial stage of protein oligomerization and disintegrates preformed tau filaments [83]. Curcumin also decreased the soluble tau dimers and elevated heat shock proteins involved in tau clearance in transgenic mice [193]. The anti-amyloid activity of curcumin against tau is facilitated by protein-specific regions: (i) molecular dynamics simulation of curcumin interaction with a short segment of tau protein VQIVYK suggest that curcumin induce conformational changes in the oligomer aggregate, which disrupt amyloid H bonding; (ii) the presence of curcumin leads to a loosely packed fibrillar structure where two of four β-sheets dissociate; (iii) the binding of curcumin is driven presumably by Van der Waals interactions [141]. The molecular docking analysis of the interactions in the curcumin–tau complexes indicates that curcumin undergoes hydrogen bonding with the Val 337 or Ser341 residues, while the oxygen atoms of the methoxy groups bind electrostatically with Val337 and Ser 341 [194]. Curcumin has the ability to also prevent α-synuclein aggregation. Using the in vitro model of α-Syn aggregation, Pandey et al. demonstrated that curcumin inhibits α-syn aggregation in a dose-dependent manner, increases α-Syn solubility, and reduces protein aggregation in SH-SY5Y cells [195]. Singh et al. showed that (i) curcumin reduces amyloid toxicity by binding to protein oligomers and fibrils, but not monomers; (ii) the magnitude of curcumin binding to the α-Syn depends on the protein oligomerization ratio; (iii) curcumin alters the morphology of the protein oligomers; (iv) curcumin modifies the α-synuclein morphology without disintegrating them to monomers; and (v) curcumin affects the fibrillar structure by reducing its exposed hydrophobic surface [196]. Another recent study indicates that curcumin strongly inhibits α-synuclein oligomer and fibril formation by binding to the hydrophobic non-amyloid-β region by allowing a faster reconfiguration rate [197]. In addition, Gautam et al. demonstrated that an optimized cocktail of curcumin and β-cyclodextrin at a low concentration not only inhibits α-synuclein aggregation but also disaggregates preformed aggregates [21]. A molecular dynamics simulation study of curcumin with quintuplet formed from the hydrophobic central domain of α-syn responsible for protein aggregation reveals that curcumin reduces the structural stability of α-synuclein oligomers by disturbing their β-sheet structure, presumably through van der Waals and electrostatic interactions [22].

Recent evidence indicates that curcumin may possess an anti-diabetic effect by modifying IAPP misfolding [35]. More specifically, it was demonstrated that curcumin (i) modulates IAPP self-assembly by preventing the formation of helix–helix interactions and slowing down the conversation of IAPP monomers to the β-sheet structures [200,201,202]; (ii) stabilizes small-molecular-weight IAPP off-pathway oligomers [65,203]; and (iii) suppress the amylin aggregation process by blocking B-sheet conversation into less accumulation-prone secondary structures [203]. Curcumin exerts its anti-amyloid effect against IAPP aggregation, presumably through non-covalent interactions [65]. The discrete molecular dynamics simulation demonstrated that curcumin molecules, associating in the nucleation site with the exposed hydrophobic and hydrogen-bonding contacts, inhibit IAPP aggregation by stabilizing small-molecular-weight IAPP off-pathway oligomers and reducing the inter-peptide association [65]. Nedumpully-Govindan et al. highlighted the key interactions that mediate the suppression of amylin self-association by curcumin: (i) the hydrogen bonding interactions between 4-hydroxy-3-methoxy phenyl substitutions on curcumin and the backbone polar residues of amylin; (ii) π-π stacking interactions between the phenyl ring of curcumin and the aromatic side chains residues of the protein; and (iii) hydrophobic interactions with residues Leu12, Phe15, His18, Phe23, Leu27, and Tyr37 [65].

**Resveratrol**, a natural polyphenolic phytoalexin mainly found in grapes and red wine, has also been reported to exhibit excellent anti-aggregation properties towards amyloidogenic proteins [204]. More specifically, Feng et al. demonstrated that resveratrol could dose-dependently inhibit Aβ42 fibril formation and cytotoxicity but could not prevent Aβ42 oligomerization [205]. Through this study, resveratrol may impede Aβ42 interstrand hydrogen bond formation, thereby preventing Aβ42 fibrillization [205]. Moreover, Western blot results showed that Aβ42 fibrils in the presence of resveratrol disaggregated to numerous oligomers [205]. Ladiwala et al. demonstrated that the revesterol–Aβ fibrillation pathway is diverted to an off pathway, a less toxic product consisting of spherical amorphous oligomers with a dominant secondary structure of random coils [72]. The all-atom molecular dynamics simulations on Aβ42 dimers and protofibrils in the presence of resveratrol reflected its ability to interact with Aβ42 monomers, inhibiting the dimerization of Aβ42 and disrupting Aβ42 protofibrils [206]. The polyphenolic compound interacts with the residues whose side chains point inwards from the surface of the protofibril, mostly via π–π stacking interactions which presumably disrupt the β-sheet structure and K28–A42 salt bridges in the Aβ42 protofibrils [206]. Resveratrol appears to be a general inhibitor as favorable inhibition was also observed with other amyloidogenic proteins, such as tau, α-synuclein, and IAPP [37,208,209,210,211,212]. More specifically, extensive studies show that resveratrol prevents amyloid formation and provides benefits in vitro and in vivo by (i) inhibiting tau aggregation and tau oligomer-induced cytotoxicity [207]; (ii) blocking the uptake of extracellular tau oligomers by N2a cells [207]; (iii) modulating the late stages of aggregation of hyperphosphorylated tau, thus decreasing tangles and leading to the accumulation of relatively more soluble forms of hyperphosphorylated tau [37]; (iv) suppressing the aggregation of α-synuclein in a concentration-dependent manner and inhibiting synuclein-induced neurotoxicity [208,209]; (v) reducing α-synuclein aggregates and oligomer levels in the brains of A53 α-synuclein mice [208]; (vi) suppressing IAPP aggregation in the early stages in vitro and in the presence of the aggregation-fostering lipid membranes [210,211,212]; and reducing the IAPP-induced cytotoxicity on INS-1E cells [211]. Numerous studies indicate that resveratrol exerts its anti-amyloid effect presumably through nonspecific interactions [202,213,215]. According to atomistic DMD simulations by Nedumpully-Govindan et al., resveratrol reduces the self-association of IAPP by stabilizing IAPP oligomers of small molecular weights through hydrogen bonds, π-π stacking, and hydrophobic interactions [202]. The replica-exchange molecular dynamics simulations study of the early stages of human islet amyloid polypeptide segment 22–27 aggregation in the presence of the small-molecule inhibitor resveratrol indicates that IAAP hinders the fibrillation process by blocking the lateral growth of single-layered β-sheet oligomers [214]. The reduction in the overall aggregation level of IAPP was assumed to be caused by the hydrophobic and aromatic interactions of the resveratrol protein, leading to the blocking of intersheet side-chain stacking (especially stacking of the aromatic rings) between protein molecules [214]. The dominant role of the non-polar interactions in resveratrol protein inhibition was also confirmed by the all-atom molecular dynamics simulations with the hIAPP1–37 pentamer [212]. More specifically, it was demonstrated that polyphenolic compound could interrupt the structural stability of the hIAPP1–37 pentamer by reducing both the β-sheet content and the order degree of the hIAPP1–37 pentamer binding to site II of the hIAPP1–37 pentamer through hydrogen bond interactions with the Arg11 and Ala13 residues and the stronger van der Waals interactions with the aromatic rings of an inhibitor and Arg11 [212]. Moreover, a mutational analysis of the ability of resveratrol to inhibit IAPP aggregation demonstrated the predominant role of the N-terminus and Arg-11 in polypeptide–resveratrol interactions [213].

**Figure 2:**
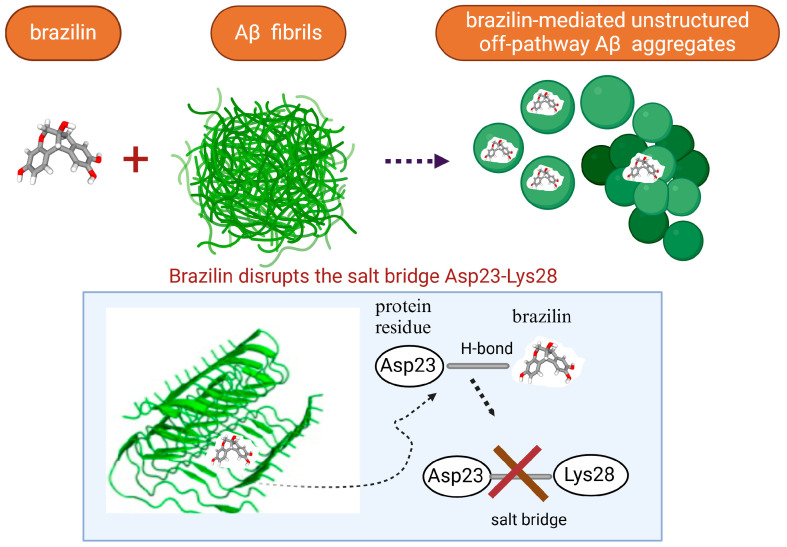
Molecular mechanism of Aβ fibril disaggregation by brazilin. Created in BioRender at https://BioRender.com/sxrz9br (accessed on 21 March 2025).

Recent studies have shown that **brazilin**, a natural polyphenolic compound extracted from *Caesalpinia sappan*, serves as an effective inhibitor of protein fibrilization, disassembles the preformed amyloid fibrils, and reduces the amyloid-induced cytotoxicity of different amyloidogenic proteins [70,214,215,216,217]. More specifically, Du et al. observed that brazilin inhibits Aβ42 fibrillogenesis with the IC50 of 1.5 μM, a value comparable with those obtained for flavonoids [70]. Moreover, brazilin effectively eliminated mature Aβ42 fibrillar aggregates [70]. Experimental evidence demonstrated that brazilin redirected protein monomers and their mature fibrils into unstructured high-molecular-weight (molecular weights of above 70 kDa) Aβ aggregates with some b-sheet structures (Figure 2) [70]. The remodeling effect of brazilin to block the formation of on-pathway toxic oligomers is believed to reduce the cytotoxicity of Aβ42 fibrils in SH-SY5Y cells [70]. Molecular dynamics studies indicate that brazilin inhibited the aggregation of Aβ42 by forming hydrogen bonds with the protein region containing the salt bridge Asp23-Lys28 [70]. Brazilin can also prevent α-synuclein aggregation [216,217]. A recent study utilizing the biochemical, biophysical, cellular, and molecular simulations studies to investigate the impact of brazilin on α-synuclein fibrillization showed that the polyphenolic compound interferes with the nucleation phase of α-synuclein aggregation in a concentration-dependent manner [216]. Moreover, through an analogy with the Aβ42 protein, these data indicate that brazilin disrupts and remodels the mature fibrils of α-synuclein into some off-pathway aggregates [216]. Anti-amyloid activities of brazilin are facilitated by the favorable hydrophobic interactions of brazilin molecules with three regions of the α-syn protein: region I, including the residues T64–G68, F94, and V95; region II, including residues H50–A53, and region III, including residues T44–E46 [216]. Nahass et al. observed that brazilin inactivates existing seeding of α-syn by stabilizing large protein aggregates [217]. The ability of brazilin to inhibit IAPP aggregation related to a reduction in the protein transition from the α-helical to the β-sheet-rich structure [218]. Brazilin protein binding with the turn region and N-terminal region of IAPP through the hydrophobic and electrostatic interactions with the residues Asn3, Thr4, Thr9, Arg11, Asn14, Phe15, His18, Ser19, Ser20, Asn21, and Phe23 results in the strong aggregation inhibition of IAPP [217]. Moreover, it was found that brazilin disaggregates hIAPP fibrils and alleviates the cytotoxicity of hIAPP aggregates [217].

## 4. Non-Phenolic Compounds as Amyloid Inhibitors

The literature extensively reports that some naturally occurring non-phenolic compounds also exhibit the potential to interfere with protein aggregation [88,245,246,247,248,249,250,251,252,253,254,255,256,257,258,259,260,261,262,263,264,265,266,267,268,269,270,271,272,273,274,275,276,277,278,279,280,281,282,283,284,285,286,287,288,289,290,291,292,293,294,295,296]. Although the underlying mechanisms of the beneficial anti-amyloid effects of the majority of non-phenolic compounds remain poorly investigated, the representatives of saponins [245,246,247,248,249,250,251,252,253], alkaloids [69,88,254,255,256,257,258,259,260,261,262,263,264,265,266], tanshinones [267,268,269,270], vitamins [271,272,273,274,275,276,277,278,279,280,281,282,283,284,285,286], and tetraterpenoids [287,288,289,290,291,292,293,294,295,296] were recently identified as promising in vivo and in vitro modulators of protein fibrillization.

**Saponins**, a broad class of amphipathic glycosides obtained from numerous plants, were found as potent amyloid inhibitors [246,247,248,249,250,251,252,253]. Namely, one member of the saponin family, bacoside A, exerted significant effects upon Aβ42 fibrillation and amyloid-induced cytotoxicity [246]. More specifically, it was suggested that the inhibition of Aβ42-induced toxicity by bacoside A was connected with its ability to suppress the transformation of Aβ42 oligomers to mature fibrils in the presence of lipid membranes [246]. Frondoside A, a saponin isolated from the sea cucumber *Cucumaria frondosa* at a low concentration, significantly delays the paralysis caused by Aβ aggregation and protects against Aβ-induced toxicity in transgenic worm tissues by reducing the level of protein oligomers [247]. Moreover, frondoside A attenuates α-synuclein aggregation in the *C. elegans* model of Parkinson’s disease [248]. Zhi et al. demonstrated that the protective effect of notoginsenoside R1 on an APP/PS1 double-transgenic mouse model of Alzheimer’s disease was connected with its ability to inhibit Aβ accumulation [249]. Onjisaponin B, mainly extracted from *Radix Polygalae*, has inhibited α-synuclein oligomerization in PC-12 cells [250]. The triterpenoid saponins from the cactus *Polaskia chichipe* are effective inhibitors of amyloid β aggregation and protect SH-SY5Y cells against aβ-associated toxicity [251]. Ginsenosides Rb1 recommended itself as a promising inhibitor of α-synuclein and aβ aggregation and toxicity [68,252]. Rb1 suppresses seeding polymerization and disaggregates the α-synuclein amyloid aggregates, presumably stabilizing the protein oligomers [68]. Similarly, ginsenoside F1 protects against Aβ aggregation in vivo and in vitro [253].

**Alkaloids**, nitrogen-containing organic compounds extracted from different medicinal plants, have received extensive attention as potent amyloid inhibitors [69,88,254,255,256,257,258,259,260,261,262,263,264,265,266]. More specifically, the isoquinoline alkaloids from the roots of *Zanthoxylum rigidum* demonstrated the moderate inhibition of Aβ aggregation on the stage of protein oligomerization [256]. Another representative of benzylisoquinoline alkaloids, berberine, was found to inhibit Aβ aggregation [257]. The berberine derivatives’ anti-amyloid activity has also been evaluated [258,259]. Caffeine, a purine plant alkaloid mainly derived from coffee beans, was shown to significantly decrease the level of Aβ amyloid and prevent the formation of amyloid plaques in the brain and blood of AD transgenic mice [260,261]. The beneficial effects of coffee consumption on type 2 diabetes mellitus were found to be connected with the ability of coffee components (caffeine, caffeic acid, and chlorogenic acid) to inhibit the formation of toxic hIAPP aggregates [226]. The molecular dynamics simulation study indicates that the self-aggregation of the caffeine molecules around hydrophobic protein residues can be crucial for caffeine anti-amyloid activity [262]. Another representative of the alkaloid family, nicotine, inhibits β-amyloidosis of Aβ(1–42) by binding to the α-helical protein structure [263]. Nicotine also exerts a beneficial effect on the aggregation of α-synuclein by increasing the lag time of protein nucleation and reducing the formation of toxic oligomeric species in a concentration-dependent manner [264,265]. Galantamine, an alkaloid compound isolated from *Galanthus woronowi*, suppresses the fibrillation and β-amyloid-induced cytotoxicity in SH-SY5Y human neuroblastoma cells [266].

**Tanshinones**, lipophilic compounds extracted from the traditional Chinese medicinal herb *Salvia miltiorrhiza*, have been reported to exhibit excellent inhibitory properties towards the aggregation of various amyloidogenic properties [267,268,269,270]. Tanshinone I and Tanshinone IIA were assayed for their influence on the Aβ42 fibrillation pathway [267]. Atomic force microscopy and ThT fluorescence results demonstrate that both compounds suppress unseeded amyloid fibril formation and disaggregate the Aβ amyloids, with the inhibitory potential being significantly higher for Tanshinone I [267]. The anti-amyloid efficacy of tanshinones was driven by their binding to the hydrophobic β-sheet groove formed by the C-terminal residues of I31-M35 and M35-V39 [267]. It was shown that Tanshinone I and Tanshinone IIA also inhibit the fibrillation process of hIAPP, changing the fibrillation pathway to the formation of amorphous aggregates by binding to the hIAPP β-sheet, thereby preventing the lateral association of hIAPP [268]. Moreover, tanshinones protect cells from Aβ- and hIAPP-induced toxicity [267,268]. Tanshinone IIA showed a strong inhibition of heparin-induced aggregation of Tau proteins [269]. Ji et al. tested the potential of tanshinones to interfere with α-synuclein aggregation in vitro and in vivo [270]. They demonstrated that Tanshinone I and Tanshinone IIA delay the secondary structural transition of proteins, alleviate the oligomerization and fibrillation of α-synuclein, and attenuate the α-synuclein amyloid formation-induced membrane damage in vitro [270]. Moreover, it was observed that tanshinone treatment prolonged the transgenic *C. elegans* NL5901 life span by reducing the aggregation of α-synuclein without affecting its expression level [270].

The prospect that the intake of certain **vitamins** may confer protection against protein-misfolding diseases has drawn substantial attention during the last decade. These essential nutrients appear to interfere with amyloid pathologies [271,272,273,274,275,276,277,278,279,280,281,282,283,284,285,286]. Namely, the administration of cholecalciferol (vitamin D3) and vitamin E compounds as a part of a vitamin-enriched diet appears to reduce fibrillar Aβ plaque deposits in the hippocampus and cortex of AD transgenic mice [272,273]. Vitamin A was demonstrated to inhibit Aβ fibrillation, destabilize preformed Aβ fibrils, and dose-dependently inhibit the oligomerization of Aβ40 and Aβ42, presumably through specific binding to the C-terminal protein region [274,275]. Moreover, vitamin A protects the human neuroblastoma cell line (SH-SY5Y) against amyloid-induced cytotoxicity through the modification of the amyloid fibrillation pathway towards the formation of non-toxic aggregates [276]. Vitamin B12 has been documented to provide beneficial anti-amyloid effects by (i) inhibiting amyloid aggregation of Aβ in a concentration-dependent manner [277,278,279]; (ii) protecting human neuronal cells against Aβ-induced cytotoxicity [277]; (iii) delaying the conformational transition of α-synuclein to the β-sheet conformations, disassembling preexisting mature α-synuclein fibrils and attenuating their cytotoxicity [280]; and inhibiting tau fibrillization to the SDS soluble oligomers via binding to cysteine residues of tau [281]. Vitamin K has been shown to influence the mechanisms involved in neurodegenerative disease pathogenesis, including protein aggregation and protein-induced neurotoxicity [282,283,284]. More specifically, vitamins K1 and K2 delay α-synuclein fibrillization to the short, sheared fibrils and amorphous aggregates by binding to the specific N-terminal site of the protein involving the residues Gly31/Lys32 [283]. Similarly, vitamin K possesses inhibitory effects against Aβ42 aggregation and amyloid-induced cytotoxicity through the modification of the protein fibrillation process to the formation of non-toxic aggregates [284]. Folic acid appeared to be an effective inhibitor of human amylin and tau protein aggregation [285,286]. Namely, folic acid stabilizes the native state of tau by directly interacting through hydrophobic forces with the protein, thereby limiting tau–seed oligomerization and consequently decelerating the polymerization of tau amyloid aggregates [285].

**Carotenoids** are another type of plant secondary metabolites reported to be effective suppressors of amyloid protein aggregation [287,288,289,290,291,292,293,294,295,296]. One representative of apocaretonoids, crocin (a carotenoid found in saffron), could modulate the fibrillation of amyloidogenic proteins by (i) inhibiting and disrupting the aggregation of Aβ42 by decreasing the exposed hydrophobic area of the protein, thereby modulating the transition from α-helical to β-sheet-rich structures [287]; (ii) redirecting the α-synuclein aggregation pathway towards the formation of off-pathway aggregates, thereby stabilizing the protein by binding to the C-terminal and central hydrophobic region domain of the E46K α-synuclein [288,289]; (iii) disassembling mature α-synuclein fibrils into seeding-incompetent intermediates [289]; (iv) suppressing the formation of tau protein filaments during the nucleation phase [290]; and (v) inhibiting the aggregation of hIAPP in a dose-dependent manner [291]. In addition to crocin, other dietary carotenoids, such as astaxanthin, fucoxanthin, cryptocapsin, etc., have recently begun to be investigated for their potential anti-amyloid effects [292,293,294,295,296]. In particular, Katayama et al. demonstrated that the carotenoid fraction from apricot fruits exhibits potent anti-amyloidogenic and fibril-destabilizing effects in vitro [292]. The keto k-ring carotenoid cryptocapsin inhibits Aβ aggregation in a dose-dependent manner through the modulation of the aggregation pathway and the disruption of Aβ aggregates [293]. Recently, many groups have reported that fucoxanthin is superior to other carotenoids in producing neuroprotective effects via multiple molecular targets, including the inhibition of amyloid protein misfolding and aggregation [294,295,296]. More specifically, it inhibits in vitro Aβ oligomer formation (at the 0.1–1 μM concentration range) and Aβ fibril formation (at 0.1–30 μM) by directly binding to the Aβ1-42 peptide through presumably hydrophobic interactions [294,295]. Furthermore, fucoxanthin might prevent Aβ-induced toxicity by inhibiting Aβ aggregation and reducing Aβ neurotoxicity in vivo [294].

## 5. Plant Extracts as Inhibitors of Amyloid Aggregation

In recent decades, there has been a growing demand for the usage of natural extracts from traditional medicinal plants for the prevention and treatment of protein-misfolding diseases. Plant extract consumption as a part of a healthy diet was shown to possess a beneficial effect in improving cognitive function and memory in individuals with neurodegenerative diseases. Numerous studies have demonstrated that natural plant extracts possess a neuroprotective role via antioxidation, antineuroinflamantory, targeting neurotransmission, and protein aggregation modulation, to name only a few. The plant extracts, which were shown to possess an anti-amyloid effect against the amyloid-β peptide; microtubule-associated protein tau; and α-synuclein and human islet amyloid polypeptide, targeting different aggregation and fibrillation stages in various experimental models, are presented in Table 3.

Recent studies indicate that *Crocus sativus* extract and its multiple constituents are especially promising for the prevention and treatment of protein-misfolding diseases [287,288,289,290,291,311,312,313]. The *Crocus sativus* L. extract not only dose-dependently inhibits protein aggregation in vivo but is also effective in fibril dissociation [311,312]. Moreover, Batarseh et al. demonstrated the reduction in the total Aβ and Aβ oligomer levels in the brains of 5XFAD mice fed with a *Crocus sativus* extract-enriched diet [313]. The authors hypothesized that the reduction in Aβ levels and plaque deposits could be in part connected with the enhanced Aβ clearance across the blood–brain barrier, the upregulation of the Aβ-degrading enzyme, and the ApoE–clearance pathway [313]. Kumar et al. reported on the effects of the plant extract of *Withania somnifera* against Aβ aggregation [340]. The results suggested that the water extract of *Withania somnifera* inhibits protein aggregation in vitro and reduces the amount of mature amyloid fibrils clustered around cholesterol microcrystals in cholesterol-promoted Aβ fibrillogenesis [340]. The 30 days course of oral administration of a semipurified extract of *Withania somnifera* was reported to inhibit the aggregation of Aβ proteins in the brains of middle-aged and old APP/PS1 Alzheimer’s disease transgenic mice through enhanced clearance of the protein [341]. More specifically, a 30 d course of treatment led to the elimination of amyloid plaques in the cortex hippocampus of middle-aged mice and a substantial reduction in protein deposits in old mice [342]. In both (middle and old) groups of mice, *Withania somnifera* significantly diminished the accumulation of β-amyloid peptides (Aβ) and oligomers in the brains [341]. Numerous studies have reported that *Withania somnifera* interventions can be beneficial in the treatment of Parkinson’s disease [342]. The methanolic extract of *Withania somnifera* was found to inhibit α-synuclein aggregation in the *Caenorhabditis elegans* NL5901 strain, although the mechanism of phytoextract actions is unknown [343].

The ability to interfere with α-synuclein aggregation was also observed for Ocimum sanctum extract [327]. The presence of an equimolar concentration of the extract was shown to attenuate the nucleation and fibril elongation process through the prevention of hydrophobic patch formation in α-synuclein [327]. Kleawyothatis et al. demonstrated that Holothuria scabra extracts possess a neuroprotective effect in the *C. elegans* model of Alzheimer’s disease by attenuating amyloid beta aggregation, reducing protein oligomers, and modulating amyloid-induced toxicity [323]. Several recent studies have demonstrated that Ginkgo biloba extract can provide protection against Aβ-induced neurotoxicity by hindering various Aβ induced events, including protein aggregation, the attenuation of Aβ oligomer formation, the accumulation of reactive oxygen species, and the production of Aβ in the brain, to name only a few [318,319,320]. Fuentes et al. reported on the effects of ethyl acetate extracts from 27 vegetable species against IAPP aggregation [304]. The authors selected *Thymus vulgaris*, *Mentha sachalinensis*, *Mentha piperita*, and *Capsicum annuum* as the most promising inhibitors of IAPP aggregation since these extracts not only inhibit protein aggregation in vivo but also protect HeLa cells from IAPP-induced toxicity [304]. Similarly, the methanolic extract of *Washingtonia filifera* seeds attenuates IAPP aggregation in a concentration-dependent manner, with complete protein inhibition at an IAPP:extract ratio of 1:5 [339].

*Bacopa monnieri* extract is a rich source of different phytochemicals, including saponins, alcohols, steroids, alkaloids, flavonoids, glycosides, and cucurbitacins, and has been reported to possess neuroprotective properties against various neurological disorders [298,299]. More specifically, the plant extract of *Bacopa monnieri* has been shown to suppress amyloid aggregation of the Aβ [298] and tau [301] proteins in vitro and reduces the tau-mediated toxicity in tau-stressed cells [301]. The short-term and long-term administration of this extract as a treatment for PSAPP mice significantly reduced the Aβ42 and Aβ40 amyloid levels in the cortex [299]. Additionally, *Bacopa monnieri* extract protects neurons from beta-amyloid-induced cell death [300]. Jadiya et al., utilizing the transgenic and pharmacological *Caenorhabditis elegans* models of Parkinson’s disease, demonstrated that treatments of worms with *Bacopa monnieri* extract led to a significant reduction in α-synuclein aggregation in NL5901 nematodes [301]. The anti-amyloid activity against Aβ and synuclein aggregation was also observed for *Centella asiatica* plant extracts [298,306,307]. The water extract of *Centella Asiatica* completely inhibited α-synuclein aggregation regardless of the stage of protein fibrillation and is capable of dissociating the mature amyloid fibrils [307]. Similarly, an ethanolic extract of *Geum urbanum* inhibits α-synuclein fibrillation by either decreasing the fibrillation capability of one or more of the intermediate states prone to aggregation or by guiding α-synuclein aggregation toward a non-fibrillar state [317].

Dhouafli et al. demonstrated that plant extracts of *Lawsonia inermis*, *Punica granatum*, and *Pistacia lentiscus* could serve as potent neuroprotective therapeutic agents against Alzheimer’s disease [325]. The methanolic extracts of these plants, especially *Lawsonia inermis*, appeared to be effective suppressors of Aβ1-42 aggregation in the early stages of β-sheet-rich structure formation, thus disfavoring the appearance of toxic protein oligomers [325]. Moreover, *Lawsonia inermis* reduced amyloid-induced cytotoxicity in the human neuroblastoma SH-SY5Y cells by inhibiting the interaction between Aβ aggregates and plasma membranes [325]. Liao et al. suggested the potential effectiveness of *Glycyrrhiza uralensis* for Parkinson’s disease treatment [321]. They demonstrated that the ethanolic extract of *Glycyrrhiza uralensis* and its major phytochemicals isoliquiritigenin and liquiritin reduce amyloid aggregation and amyloid-mediated toxicity in vitro and in the *C. elegans* NL5901 model [321].

A recent study reported the ability of *Allium roseum* L. ethanolic extract to interfere with aβ-42 fibrillogenesis [296]. More specifically, it was observed that *A. roseum* extract dose-dependently suppresses Aβ-42 fibrillation by modulating the structure of aggregates to an amorphous one [296]. Moreover, *A. roseum* extract reduces amyloid-induced cytotoxicity toward the human neuroblastoma cell SH-SY5Y by inhibiting aggregates binding to the cell membranes and also protects aggregate-exposed cells by counteracting the oxidative stress through a reduction in ROS and free intracellular Ca^2+^ levels [296]. The dichloromethane and n-butanol extracts of *Scutellaria pinnatifida* were found to strongly inhibit α-synuclein aggregation and protect PC12 cells and dopaminergic neurons from amyloid-induced toxicity, presumably by attenuating synuclein oligomers [335]. Ogara et al. evaluated the Aβ aggregation inhibitory activity of 11 seaweed ethanolic and boiling water extracts, including *Cystoseira hakodatensis*, *Sargassum horneri*, *Sargassum fusiforme*, *Saccharina japonica*, *Saccharina sculpera*, *Undaria pinnatifida*, *Alaria crassifolia*, *Mazzaella japonica*, *Chondrus yendoi*, and *Gloiopeltis furcata* [295]. They demonstrated that the plant extracts reduce amyloid protein fibrillation and modulate fibril morphology, with the magnitude of effect higher for water boiling extracts [295].

Fujiwara et al. selected the *Uncaria rhynchophylla* extract as the most potent inhibitor of amyloid fibrillation among the several Chinese medicinal herbs tested in their study [336]. The *Uncaria rhynchophylla* extract not only reduced fibril formation in a concentration-dependent manner but also possessed a destabilizing effect on preformed Aβ fibrils [336]. Similar dual inhibiting/disaggregating effects were observed for Cinnamon aqueous extract in the presence of the tau protein [307]. The *Uncaria tomentosa* extract was identified as a potential inhibitor for both brain Aβ “plaques” and tau protein-containing neurofibrillary “tangles” [337]. More specifically, the *Uncaria tomentosa* extract and its polyphenolic PTI-777 fraction not only appeared as effective reducers of Aβ 1–40 amyloid fibril and tau protein paired helical filament/fibril formation but also disassembled preformed aggregates [337]. A direct infusion of a polyphenolic fraction of *Uncaria tomentosa* extract into the cortex of 8-month-old TASD-41 APP transgenic mice during a 14-day treatment was followed by a marked reduction in the number of amyloid plaques in the hippocampus and cortex [337]. Moreover, the major component of *Uncaria tomentosa* PTI-777 can cross the blood–brain barrier and reduce brain plaque load within 30 days of peripheral administration in transgenic mice [337].

*Rosa Damascena*, a medicinal herb containing powerful antioxidants and bioactive secondary metabolites, was found to interfere with the fibrillation process of synuclein and IAPP [333]. More specifically, the methanolic extract of *Rosa damascena* showed a concentration-dependent α-synuclein aggregation inhibition, shifting the early stages of the protein fibrillization process to the formation of off-pathway less toxic protein oligomers [333]. The *Rosa damascena* extract also protects SH-SY5Y cells from α-synuclein-induced toxicity and modulates the aggregation of the IAPP protein [334]. The ability to interfere with protein aggregation was also reported for the methanolic extract of walnut [324]. The walnut extract not only inhibited Aβ fibrillation but was also able to dissociate the preformed fibrils [324].

## 6. Conclusions

Protein-misfolding diseases are among the leading causes of morbidity and mortality, particularly among the elderly, and have historically been regarded as conditions with limited therapeutic options [343]. The primary culprits behind the pathological events in protein-misfolding diseases are misfolded protein aggregates, which contribute to disease progression through mechanisms such as toxicity, improper localization, degradation, and amyloid accumulation, ultimately leading to cell damage and impaired functionality [344]. To develop effective therapeutic strategies against amyloid-associated diseases, it is important to elucidate the molecular mechanisms underlying protein misfolding and aggregation, as well as the factors involved in amyloid-induced toxicity. To date, one of the primary therapeutic strategies against protein-misfolding diseases (PMDs) has focused on inhibiting the aggregation of amyloidogenic proteins and remodeling toxic protein aggregates into non-toxic intermediates [21,22,23,343].

Plant extracts, extract-based formulations, and plant-derived phytochemicals have been shown to effectively target amyloid-associated proteins by modulating the aggregation process and disaggregating preformed fibrillar structures in both in vitro and in vivo models. A key advantage of plant-based formulations over synthetic amyloid-inhibiting substances lies in their dual role: they function as nutraceuticals, contributing to a healthy diet, while also serving as pharmaceuticals when administered at higher concentrations. Our study demonstrated the potential of at least 59 plant extracts to target different aggregation and fibrillation stages of the amyloid-β peptide, microtubule-associated protein tau, α-synuclein, and islet amyloid polypeptide in various experimental models. Most of these studies have focused on assessing the inhibition potential of extracts or extract fractions, while information regarding the specific active compounds responsible for the observed anti-amyloid effects remains largely unknown. Given the intricate stereochemistry of plant-based compounds, their complete synthetic replication remains a significant challenge. Moreover, we believe that the search for effective anti-amyloid compounds requires an integrated approach that combines bioprospecting, advanced extraction engineering, metabolomics, isolation, purification, and bioactivity assessments. The importance of such studies is underscored by the fact that compounds within extracts may exert both antagonistic and synergistic effects.

Most of the compounds reviewed exhibited promising anti-amyloid effects against multiple amyloid proteins, making them strong candidates for further optimization in the treatment of various protein-misfolding diseases. Additionally, the presented data highlight existing gaps in research, as some natural compounds have only been tested against a single amyloid protein, leaving their broader inhibitory potential unexplored. It is important to note that the reviewed compounds do not exhibit uniform efficacy across different amyloidogenic proteins, as their mechanisms of action vary significantly depending on the specific protein involved. Notably, although several studies have demonstrated the potential of numerous non-phenolic compounds in modulating the aggregation pathway of the amyloid-β peptide, microtubule-associated protein tau, α-synuclein, and human islet amyloid polypeptide, these compounds remain less explored compared to phenolic compounds. To the best of our knowledge, crocin is the only representative compound tested for its ability to suppress the aggregation process of all the reviewed proteins, while the potential of other compounds has primarily been investigated for a single protein. Moreover, the molecular mechanisms underlying non-phenolic amyloid inhibition have been studied to a lesser extent. Therefore, large-scale structure–activity relationship studies should be conducted using standardized methodologies and assay conditions to determine the role of phytochemicals and protein structural factors in protein aggregation, with the ultimate goal of developing effective strategies to prevent protein-misfolding diseases.

The availability of big data in online databases on the potential compounds to inhibit protein aggregation has led to the emergence of artificial intelligence (AI) techniques for analyzing and predicting effective anti-amyloid agents in the last few years. Machine learning models such as AMYLOGRAPH, Aggrescan3D, ZipperDB, PyRMD, and machine-learning-enhanced molecular docking trained on large datasets of protein structures and interactions can predict aggregation-prone sequences and screen for potential inhibitors with high accuracy [345,346,347,348]. Screening different plant compounds and their numerous isomers, which can even be unidentified in the plant extracts, using AI-based approaches is a promising strategy to identify effective and selective aggregation inhibitors, offering promising advancements in protein-misfolding diseases.

Many plant-derived compounds, particularly polyphenols, have been tested in preclinical animal models [245] and clinical human trials, including epigallocatechin gallate (EGCG) [349], resveratrol [350], and curcumin [351]. Despite encouraging in vitro and in vivo results, these natural inhibitors have had limited success in clinical trials due to their poor metabolic stability and low bioavailability at pharmacologically relevant concentrations [349,350,351,352]. Researchers have attempted to overcome these limitations using nanocarriers and nanotechnology to improve bioavailability, potentially enhancing clinical efficacy. Additionally, further research is needed to address concerns regarding the use of phytochemical-loaded nanocarriers in targeting protein aggregation in protein-misfolding diseases.

Overall, while significant progress has been made in identifying natural compounds with anti-amyloid potential, further studies are essential to fully understand their mechanisms of action, improve their bioavailability, and assess their therapeutic efficacy in clinical settings.

## Figures and Tables

**Table 1:** Flavonoids as inhibitors of the Aβ peptide, tau protein, synuclein, and IAPP fibrillation.

Plant-Based Compound	Main Source	Targeted Protein	IC_50_	Effects	References
(−)Epigallocatechin-3-gallate (EGCG) 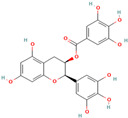	Green tea	Aβ	15 µM (Aβ42) 3 µM (Aβ40)	Disrupts and remodels the mature amyloid fibrils and protein-toxic oligomers into non-fibril structures through the non-covalent binding mechanism (all proteins) and the formation of covalent Schiff base adducts (Aβ1–40 and IAPP8–24); Prevents the early steps of protein self-aggregation.	[58,81,100,101,102,103,104,105,106,107,108,109,110,111,112,113,114,115,116]
		Tau	Nd **˟**		
		synuclein	Nd		
		IAPP	Nd		
Baicalein 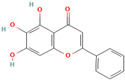	*Scutellaria baicalensis*	Aβ42	1.35 μM	Inhibits the formation of amyloid fibrils in the initial stages of protein aggregation through covalent adduct formation; Inhibits and stabilizes protein oligomers; Disaggregates the preformed amyloid fibrils into the fragments.	[80,89,90,91,117,118,119,120,121,122,123,124]
		Tau	35.8 μM		
		synuclein	Nd		
		IAPP	92.1 μM		
Quercetin 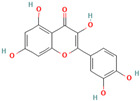	fruits, vegetables, red wine	Aβ42	15.3 μM	Inhibits fibril growth and disaggregates the performed amyloid fibrils of Aβ and IAPP via the covalent interaction mechanism at the early stages of their aggregation; Suppresses the fibrillation of α-synuclein and disaggregates preformed fibrils by inhibiting the fibril growth step; Binds β-amyloid oligomers and modifies oligomer structures; Attenuates and reverses Aβ25–35 fibril formation in vitro; Stabilizes α-synuclein oligomers.	[8,41,61,76,94,125,126,127,128,129,130,131]
		synuclein	Nd		
		IAPP	Nd		
		tau	>200 μM		
Myricetin 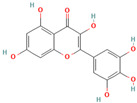	fruits, vegetables, red wine	Aβ42	15.1 μM	Inhibits the oligomerization and the statistical coils-sheet conversion of Aβ40 and Aβ42 through specific interactions with the monomeric protein region; Remodels prefibrillar oligomers and fibrils into unstructured, insoluble aggregates; Blocks tau aggregation by interfering with the elongation phase of fibril assembly; Suppresses the formation of oligomers and fibrils of α-syn by preventing its conformational change into β-sheets and disaggregates the fibrils in a dose-dependent manner; Inhibits hIAPP fibrillization and protects the mammalian PC12 cells from hIAPP-mediated cytotoxicity.	[66,71,76,94,132,133,134,135,136,137,138,139,140,141,142,143,144]
		Tau	Nd		
		synuclein	3.57 μM		
		IAPP	Nd		
Dihydromyricetin 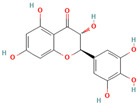	herb *Ampelopsis grossedentata*	Aβ40 Ab42	18.96 μM 25.3 μM	Inhibits the formation of Aβ40 and α-synuclein fibrils in a concentration-dependent manner by suppressing the conformational conversion of proteins to β-sheets and destroying the intramolecular hydrogen bonds through noncovalent and hydrophobic interactions with α-synuclein residues; Disassembles the preformed Aβ40 fibrils and attenuates Aβ40-induced cytotoxicity in PC12 cells; Suppresses Aβ42 aggregation through the formation of the adduct between Lys residues in Aβ42 and the o-quinone part of the flavonoid.	[94,145,146,147]
		synuclein	Nd		
Rutin 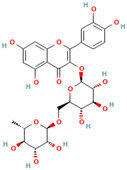	Buckwheat, citrus fruits, apicots, blackberries, apple, cherries, red wine.	Aβ, Tau, IAPP	Nd	Dose-dependent inhibition of protein fibrillization (IAPP, Aβ-42); Attenuates and reverses Aβ25–35 fibril formation in vitro; Suppresses a lag phase of IAPP fibrillization and protein misfolding; Reverts the preformed fibrillar IAPP structures into the monomeric α-helical confirmation; Stabilizes the IAPP monomer structure; Improves spatial memory in AD transgenic mice by reducing the Aβ oligomer level and attenuating oxidative stress and neuroinflammation; Inhibits tau aggregation and tau oligomer-induced cytotoxicity in a concentration-dependent manner.	[41,76,82,126,148,149,150,151]
Morin 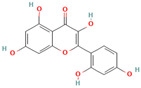	almond hulls, guava leaves, old fustic, onion, apple, tea, red wine	Aβ1-42	30.3 μM	Moderately suppresses the aggregation of Aβ-42; Decreases hyperphosphorylated tau in tangles in brain sections of transgenic mice by blocking or reversing late stages of aggregation of the protein, thus leading to accumulation of relatively more soluble forms of tau; Slightly accelerates the secondary nucleation process of α-synuclein and inhibits fibril elongation, thereby reducing the formation of α-synuclein oligomers; Interacts with hIAPP in the amyloidosis core by preventing the accumulation and twisting of fibrils and breaks down the preformed fibrils into non-toxic species; Alters the structural properties of hIAPP by binding to the amyloidogenic region of the protein through hydrogen bond, π–π, and hydrophobic interactions with the residues His18, Phe23, and Ile26.	[37,62,94,152,153,154]
		Tau	13 µM		
		synuclein	Nd		
		IAPP	Nd		
(+)-Taxifolin 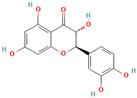	onions, milk thistle, Douglas fir bark	Aβ	33 μM	Suppresses the elongation phase of Aβ42 aggregation through the formation of the covalent adducts with the Lys 16 residue in Aβ42; Possesses potential in inhibiting and disaggregating IAPP amyloid fibrils	[63,94,131,155,156,157,158]
		Tau	>200 μM		
		synuclein	>40 μM		
		IAPP	Nd		
Rottlerin 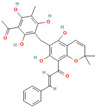	Kamala powder of the *Mallotus philippinensis* fruits	Aβ	19.55 μM	Inhibits nuclei formation or disassembles performed fibrils by targeting the amyloidogenic LVFFAED part of the Aβ1-40 peptide.	[39]
Transilitin 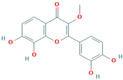	*Luculia pinceana*, *Acacia cyperophylla*	Aβ	5.4 μM	Significantly inhibits Aβ1-42 aggregation, forming hydrogen bonding primarily within their B-ring hydroxyl groups and the key Lys 16 residue; Demonstrates neuroprotective efficacy against Aβ-evoked PC12 neuronal cell toxicity; Inhibits toxic amyloidogenic aggregation-prone α-synuclein mutants by stabilizing the native unfolded αSA53T conformations;	[130,159,160]
		synuclein	Nd		
Kaempferol 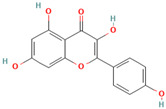	fruits, vegetables, and medicinal plants	Aβ	75.1 μM	Suppresses the aggregation of Aβ42 and destabilizes preformed amyloid fibrils; Inhibits the amyloid fibril formation of α-Syn, inducing the formation of a structure resembling an amorphous aggregation; Protects against cytotoxicity induced by Aβ42 in a concentration-dependent manner.	[94,135,161,162,163,164]
		synuclein	Nd		
Fisetin 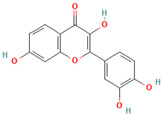	fruits and vegetables	Aβ	43.7 μM	Inhibits the β-sheet transformation of Aβ42; Binds with α-Syn and delays protein structural transitions into β-sheet-rich fibrillar structures; Inhibits tau K18 aggregation in a concentration-dependent manner, increasing the stability of certain conformations of protein by generating hydrogen bonds with K18, thereby inducing a more compact and less flexible protein structure; Depolymerizes tau aggregates after their formation; Inhibits IAPP aggregation by stabilizing the protein monomeric state; Increases the area of the aggregates and decreases the toxicity of α-syn–GFP in a yeast model expressing human αsyn–GFP.	[56,77,165,166,167]
		Tau	41.45 μM		
		synuclein	Nd		
		hIAPP	Nd		
Apigenin 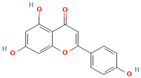	fruits and vegetables	Aβ	Nd	Suppresses Aβ40 nucleation, altering the normal fibrillation pathway and the generation of aggregates in non-amyloid conformations, and disrupts Aβ42 protofibrils from destabilizing protofibrils predominantly through electrostatic interactions; Simultaneously delays and reduces the formation of α-synuclein oligomers by stabilizing unfolded structures via hydrophobic interactions and hydrogen bonding; Protects against membrane perturbation induced by aggregated wild-type and mutant α-synuclein; Reduces Tau aggregation, oxidative stress, and caspase-1 activity as well as improves neurite outgrowth in SH-SY5Y cells;	[61,64,160,168,169,170,171]
		tau	Nd		
		synuclein	Nd		
Genistein 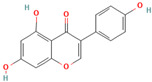	soybeans and soy products	Aβ	Nd	Inhibits the aggregation of Aβ and hIAPP from monomers to fibrils, modulating the conformational transition of monomeric Aβ and hIAPP peptides from random coils toward β-sheets; Inhibits the formation of Aβ1–40-positive aggregates in the brain tissue of rats; Possesses the potential to disaggregate the IAPP fibrils, loosening the two nearest peptide chains by binding with two regions of hIAPP, L12–A13–N14, and N31–V32–G33–S34–N35;	[82,172,173]
		hIAPP	Nd		
Icariin 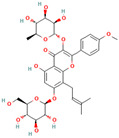	Chinese herbal medicine Herba Epimedii	Aβ	0.48 μM	Inhibits Aβ1-42 aggregation in a dose-dependent manner; Reduces the Aβ burden and amyloid plaque deposition in the hippocampus of APPV717I transgenic mice.	[174,175]
Silybin 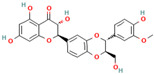	*Silybum Marianum*	Aβ	Nd	Suppresses amyloid β-protein fibril formation and neurotoxicity in PC12 in a dose-dependent manner; Stimulates an inhibitory effect on the production of a-syn fibrils by preventing a-syn, α-helix, or ß-sheet conformations; Greatly inhibits the formation of hIAPP fibrils and hIAPP oligomers by disrupting the structural transition of hIAPP monomers from a random coil to an α-helix via a strong binding of silybin to the precise hIAPP regions of the S20-S29 amyloidogenic core, the N-terminal domain.	[82,176,177,178,179,180]
		synuclein	Nd		
		hIAPP	Nd		
Luteolin 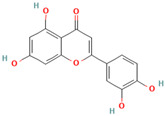	celery, parsley, peppermint, thyme, oregano	Aβ	6.4 μM	Dose-dependently inhibits Aβ aggregation by causing a significant reduction in the oligomeric species size; Interacts with Aβ40 primary oligomers, leading to the formation of conformationally distinct species; Accelerates primary nucleation events via catechol autoxidation into *o*-quinones followed by the covalent modification of lysine side chains by redirecting the oligomerization pathway towards off-pathway non-amyloidogenic species; Inhibits the early stages of α-synuclein fibrillization.	[73,92,159,181]

˟ Nd means not determined.

**Table 2:** Non-flavonoid polyphenolic compounds as an inhibitor of protein fibrillization.

Plant-Based Compound	Main Source	Targeted Protein	IC_50_	Effects	References
Curcumin 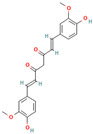	*Curcuma longa*	Aβ1-40	0.8 µM	Inhibits the aggregation of Aβ, synuclein, IAPP, and tau in the initial stages of protein aggregation through a non-covalent binding mechanism; Dose-dependently destabilizes preformed amyloid fibrils; Enriches the population of non-toxic “off-pathway” soluble oligomers and prefibrillar aggregates of Aβ and α-synuclein; Targets the cross-beta spine of amyloid fibrils and oligomers in the early stages of protein aggregation.	[35,36,65,83,182,183,184,185,186,187,188,189,190,191,192,193,194,195,196,197,198,199,200,201,202,203]
		Tau	3.0 µM		
		synuclein	Nd		
		IAPP	Nd		
Resveratrol 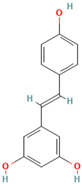	red grapes	Aβ1-42	11 µM	Dose-dependently inhibits Ab42 fibril formation and cytotoxicity, presumably by blocking inter-strand hydrogen bond formation; Selectively remodels soluble oligomers and fibrils of amyloid Aβ into off-pathway conformers; Inhibits tau aggregation and tau oligomer-induced cytotoxicity in a concentration-dependent manner; Inhibits α-synuclein aggregation and cytotoxicity in vitro in a concentration-dependent manner; Prevents IAPP fibril formation during the early stages of fibrillogenesis; Prevents IAPP fibril formation in the presence of anionic lipid membranes; Reduces the cytotoxicity of IAPP on INS-1E cells by arresting IAPP fibril formation at the early stages.	[37,72,183,190,204,205,206,207,208,209,210,211,212,213,214]
		Tau	10 µM		
		synuclein	Nd		
		IAPP	3.3 µM		
Brazilin 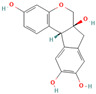	wood of *Caesalpinia echinata* or *Caesalpinia sappan*	Aβ1-42	1.5 µM	Inhibits Aβ aggregation and disaggregates the preformed amyloid fibrils remodeling monomers and mature fibrils into unstructured off-pathway high-molecular-weight aggregates via hydrophobic and hydrogen bonding interactions; Reduces Aβ42 amyloid-induced cytotoxicity in the SH-SY5Y cells; Reduces α-syn fibrillogenesis, disrupts the preformed fibrils in a concentration-dependent manner, and diminishes the cytotoxicity induced by α-synuclein aggregates; Inhibits hIAPP fibrillogenesis in a dose-dependent manner by delaying the conformational transition of hIAPP from its helical to the β-sheet form, alleviates hIAPP-induced cytotoxicity, and disassembles preexisting hIAPP fibrils.	[70,215,216,217,218,219,220,221]
		synuclein	Nd		
		IAPP	Nd		
Altenusin 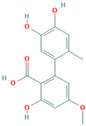	Fungal endophyte *Alternaria*	Tau	Nd	Reduces the heparin-induced polymerization of the recombinant four-repeat domain tau protein by inducing the formation of distinct globular structures instead of large oligomers	[40]
Oleocanthal 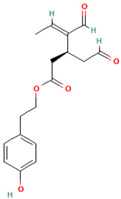	Olive oil	Aβ	2.9 µM	Changes the oligomeric state of soluble oligomers of the amyloid-β peptide with a time- and concentration-dependent trend toward higher molecular weight species; Abrogates the fibrillization of tau by locking tau into the naturally unfolded state through an adduct with lysine via initial Schiff base formation; two aldehyde groups present in oleocanthal are essential to prevent tau fibrillogenesis; Exhibits nonspecific covalent interactions with tau-441, inducing a conformational rearrangement because of a fast transition of the tau-441 secondary structure from a random coil to an α-helix.	[38,95,219]
		Tau			
			Nd		
Oleuropein aglycone 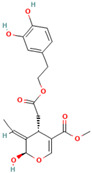	olive oil	Aβ	Nd	Interferes with the amyloid aggregation of Aβ42, skipping the appearance of toxic oligomers and favoring accumulation into off-pathway aggregates; Protects against Aβ42 aggregation and plaque formation in tissue, specifically in *C. elegans* and the TgCRND8 mouse model; Inhibits tau fibrillization to smaller fibrillar aggregates; Stabilizes α-syn oligomerization and favors the growth of non-toxic aggregates.	[67,78,220,221,222,223,224]
		Tau	1.4 μM		
		synuclein	Nd		
		IAPP	Nd		
Caffeic acid 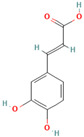	coffee beans, fruits, potatoes, carrots and propolis	Aβ	17.9 µM	Inhibits Aβ42 fibrillation and disrupts mature amyloid fibrils; Inhibits synuclein aggregation on the lag phase of protein fibrilization, promoting the formation of stable off-pathway amorphous aggregates; Disaggregates the α-synuclein amyloid fibrils to the less twisted rope-like protofilaments; Suppresses the initial oligomerization step of IAPP aggregation, disrupts the formation of fibrils, and redirects the amyloidogenic molecules into off-pathway aggregates;	[76,84,225,226,227]
		Tau	Nd		
		synuclein	Nd		
		IAPP	57.6 µM		
Tannic acid 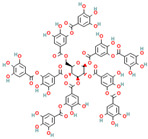	Tara pods, gallnuts or *Sicilian sumac* leaves	Aβ	0.1 μM	Inhibits Aβ amyloid assembly from the monomeric form into well-ordered fibrils; Dose-dependently inhibits the aggregation of tau peptide R3, suppressing its conformational transition from a random coil structure to a β-sheet system by forming a hairpin binding motif stabilized by hydrogen bonding, hydrophilic–hydrophobic interactions, and static electrical interactions; Reduces tau aggregation induced by fibrils of tau R3 in the human neuroblastoma cell, SK-N-SH; Inhibits the formation of αSyn aggregates in vitro and in mouse primary cultured neurons and the PD model *C. elegans*; Inhibits IAPP aggregation in vitro and IAPP-induced cellular toxicity.	[230,231,232,233,234]
		Tau	3.5 μM		
		synuclein	Nd		
		IAPP	Nd		
Ferulic acid 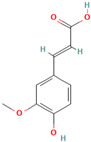	cereal grains, fruits, vegetables	Aβ	Nd	Dose-dependently inhibits Aβ1-40 and Aβ1-42 fibril formation and destabilizes preformed fibrils; Inhibits the monomer-to-oligomer transition by blocking the hydrogen bond with the forming β-sheets but accelerates the Aβ42 oligomer-to-fibril transition; Reduces Aβ42-induced neurotoxicity in SHSY5Y cells; Protects PC12 cells against oxidative damage by modulating β-amyloid aggregation; Dose-dependently inhibits the formation and extension of α-synuclein aggregation and destabilizes preformed fibrils, Inhibits IAPP fibrillation but only at high molar ratios.	[3,76,235,236,237,238,239,240,241]
		Tau	Nd		
		synuclein	13 μM		
		IAPP	40 μM		
Gallic acid 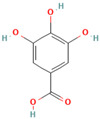	fruits, teas, cloves, and vinegars.	Aβ	Nd	Inhibits Aβ42 fibrillation in a dose-dependent manner; Prevents the aggregation of an aggregation-prone form of α-synuclein to the short amorphous aggregates by directly interacting and stabilizing the unfolded monomeric species; Inhibits the exponential phase of IAPP fibrillation by encouraging IAPP seeding but slowing down fibril growth in the elongation phase.	[76,227,228,229,230]
		Tau	92 μM		
		synuclein	Nd		
		IAPP	Nd		
Rosmarinic acid 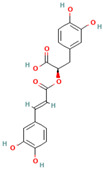	species in the Labiatae and Boraginaceae family of plants.	Aβ	4.8 μM	Suppresses Aβ aggregation and disrupts preformed Aβ fibrils by intercalating into the Aβ oligomer structure at the interface between monomers; Prevents tau assembly and diminishes the β-sheet content in a dose-dependent manner through hydrogen bond interactions with glutamine and lysine side chain groups at both sides of the steric zipper and the salt bridge between the carboxylate group of rosmarinic acid and two Lys311 ammonium ions from ^306^VQIVYK^311^ fibers; Dose-dependently inhibits the formation of α-synuclein fibrils and disaggregates the preformed fibrillar structures, presumably breaking the β-sheet form through van der Waals and electrostatic interactions; remodels amylin amyloid fibrils into non-toxic aggregates, presumably via off-pathway channels; reduces amylin amyloid deposition in the pancreas of HIP rats.	[74,199,239,242,243,244]
		Tau	7.7 μM		
		synuclein	Nd		
		IAPP	3.1 μM		

**Table 3:** Plant extracts providing anti-amyloid properties.

Plant Extract	Extract Type	Model System	Effects	References
*Acer saccharum*	acetone, water, and ethanol	in vitro, α-synuclein, and Aβ1-40	inhibits the formation of amyloid fibrils	[294]
*Alaria crassifolia*	ethanolic and boiling water	in vitro and Aβ	inhibits amyloid fibrillation and alters fibril morphology	[295]
*Allium Roseum*	ethanolic	in vitro, Aβ1-42, and SH-SY5Y cells	inhibits amyloid-β aggregation and amyloid-induced cytotoxicity	[296]
*Alisma orientale*	ethanolic	in vitro, Aβ1-42	25% of protein aggregation inhibition	[297]
*Bacopa monnieri*	aqueous	in vitro and MAβ1-40 PSAPP mice	inhibits the formation of amyloid fibrils and reduces amyloid levels in PSAPP mice	[298,299,300]
	ethanolic	in vitro, tau, and tau-stressed Neuro2A neuronal cells	inhibits tau aggregation and reduces tau-mediated toxicity in cells	[301]
		*NL5901 strain of C. elegans*	reduces synuclein aggregation in NL5901 worms	[302]
*Camellia sinensis*	methanolic	in vitro and Aβ1-42	inhibits amyloid-β aggregation	[303]
*Capsicum annuum*	ethyl acetate	In vitro, IAPP, and HeLa cells	attenuates IAPP aggregation and protects cells from amyloid-induced toxicity	[304]
*Cassi Tore*	ethyl acetate	in vitro, Aβ1-42, SK-N-SH, and SH-SY5Y cells	inhibits amyloid fibrillation in a dose-dependent manner and alleviates Aβ-induced oxidative stress in human neuroblastoma cell lines.	[305]
*Centella asiatica*	ethanolic	in vitro and MAβ1-40 PC12 cells	reduces the formation of amyloid fibrils and protects PC12 cells from amyloid-induced toxicity	[298]
	aqueous	in vitro and α-synuclein	inhibits protein aggregation and dissociates fibrils	[306,307]
*Chondrous yendoi*	ethanolic and boiling water	in vitro and Aβ	inhibits amyloid fibrillation and alters fibril morphology	[295]
*Cinnamomumverum*	aqueous	in vitro and tau^187^	inhibits tau filament formation, disaggregates preformed tau aggregates, and causes alterations in the morphology of AD brain-isolated fibrils	[308]
*Cocos nucifera*	ethanolic	*Transgenic C. elegans*	reduces Aβ deposits by 30.3% in CL2006	[309]
*Cystoseira hakodatensis*	ethanolic and boiling water	in vitro and Aβ	inhibits amyloid fibrillation and alters fibril morphology	[295]
*Cremastra appendiculata*	ethanolic	in vitro and Aβ1-42	inhibits amyloid-β aggregation	[310]
*Crocus sativus* L. *(Saffron)*	water/methanol 50:50	in vitro and Aβ1-40	inhibits the formation of amyloid fibrils in a concentration-dependent manner	[311]
	DMSO/water 10:90	in vitro and α-synuclein	dose-dependently inhibits protein aggregation and dissociates fibrils	[312]
	water/ethanol (1:1)	AD mouse model	significantly reduced (53%) the total Aβ levels in 5XFAD brains hippocampi and decreased Aβ brain deposits	[313]
*Cuminum cyminum*	Essential oil	in vitro and α-synuclein	dose-dependently inhibits protein aggregation	[314]
*Cussonia paniculata*	dichloromethane/methanol	in vitro and HeLa cells	reduces Aβ production in HeLa cells	[315]
*Curcuma longa*	methanolic	in vitro and Aβ1-42	inhibits amyloid-β aggregation	[303]
*Elettaria cardamomum*	1,8-cineole-rich extract	in vitro, Aβ1-42, and SH-SY5Y cells	prevents Aβ oligomerization and protects SH-SY5Y cells against iron-induced death	[316]
*Gardenia jasminoides*	ethanolic	in vitro and Aβ1-42	32% of protein aggregation inhibition	[297]
*Geum urbanum*	ethanolic	in vitro and α-synuclein	inhibits protein aggregation and disaggregates fibrils	[317]
*Gloiopeltis furcata*	ethanolic and boiling water	in vitro and Aβ	inhibits amyloid fibrillation and alters fibril morphology	[295]
*Ginkgo* *giloba*	standardized EGb761	in vitro, Aβ1-40, neuroblastoma cell lines, and *C. elegans*	inhibits amyloid fibrillation and prevents Aβ aggregation in the medium of Aβ-producing cells	[318,319,320]
*Glycyrrhiza uralensis*	ethanolic	in vitro, α-synuclein, and *C. elegans* NL5901	inhibits protein aggregation and alleviates amyloid-induced toxicity	[321]
*Guettarda* *speciosa*	methanol, chloroform	in vitro, Aβ1-42, and SH-SY5Y cells	reduces the formation of amyloid fibrils	[322]
*Holothuria scarba*	ethanol, buthanol and ethyl acetate	*Caenorhabditis elegans*	attenuates amyloid-β aggregation and toxicity	[323]
*Juglas regia*	methanolic	in vitro and Aβ	inhibits amyloid fibrillation and defibrillizes performed fibrils	[324]
*Juncus effusus*	ethanolic	in vitro and Aβ1-42	68% of protein aggregation inhibition	[297]
*Juncus setchuensis*	ethanolic	in vitro and Aβ1-42	68% of protein aggregation inhibition	[297]
*Lawsonia inermis* L.	methanolic	in vitro, Aβ1-42, and SH-SY5Y cells	inhibits Aβ1-42 aggregation in the early stage of β-sheet-rich structure formation and reduces amyloid-induced cytotoxicity	[325]
*Mazzaella japonica*	ethanolic and boiling water	in vitro and Aβ	inhibits amyloid fibrillation, alters fibril morphology	[295]
*Mentha sachalinensis*	ethyl acetate	In vitro, IAPP, and HeLa cells	attenuates IAPP aggregation and protects cells from amyloid-induced toxicity	[304]
*Mentha piperite*	ethyl acetate	In vitro, IAPP, and HeLa cells	attenuates IAPP aggregation, protects cells from amyloid-induced toxicity	[304]
*Nardostachys chinensi*	ethanolic	in vitro and Aβ1-42	62.8% of protein aggregation inhibition	[297]
*Nelumbinis folium*	ethanolic	in vitro and Aβ1-42	inhibits protein aggregation	[326]
*Ocimum* *sanctum*	DMSO	in vitro, α-synuclein, and mouse Neuro2a cells	inhibits protein aggregation, disaggregates preformed fibrils, and protects mouse neuroblastoma cells against α-synuclein amyloid-induced cytotoxicity.	[327]
*Olea europaea*	acetone/water	*transgenic worms*	prevents β-amyloid aggregation in *Caenorhabditis elegans*	[328]
*Origanum glandulosum*	ethyl acetate	in vitro and Aβ25-35	reduces amyloid fibril formation in a dose-dependent manner	[329]
*Panionia* *suffruticosa*	water	in vitro, Aβ1-42, Aβ1-40, in vivo, and Tg2576 mice	inhibits fibril formation, destabilizes preformed amyloid fibrils, and inhibits the accumulation of Aβ in the brain of Tg2576 transgenic mice	[330]
*Panax notoginseng*	Essential oil	in vitro and Aβ1-42	57.3% inhibition at 500 μg/mL against Aβ aggregation	[331]
*Perila frutescens*	ethanolic	in vitro and Aβ1-42	inhibits the formation of amyloid fibrils in a concentration-dependent manner	[332]
*Pistacia lentiscus* L.	methanolic	in vitro, Aβ1-42, and SH-SY5Y cells	hinders Aβ1-42 aggregation during the early secondary structure transition to amyloid	[325]
*Punica granatum* L.	methanolic	in vitro, Aβ1-42, and SH-SY5Y cells	hinders Aβ1-42 aggregation during the early secondary structure transition to amyloid	[325]
*Rheum officinale*	ethanolic	in vitro and Aβ1-42	92% of protein aggregation inhibition	[297]
*Rosa* *damascena*	methanolic	in vitro, α-synuclein, and SH_SY5Y cells	inhibits α-syn fibrillation in a concentration-dependent manner and reduces the toxicity of oligomers to SH-SY5Y cells.	[333]
	ethanolic	in vitro and IAPP	modulates IAPP aggregation	[334]
*Saccharina japonica*	ethanolic and boiling water	in vitro and Aβ	inhibits amyloid fibrillation and alters fibril morphology	[295]
*Saccharina sculpera*	ethanolic and boiling water	in vitro and Aβ	inhibits amyloid fibrillation and alters fibril morphology	[295]
*Sargassum fusitorme*	ethanolic and boiling water	in vitro and Aβ	inhibits amyloid fibrillation and alters fibril morphology	[295]
*Sargassum horneri*	ethanolic and boiling water	in vitro and Aβ	inhibits amyloid fibrillation and alters fibril morphology	[295]
*Schotia* *brachypetala*	dichloromethane/methanol	in vitro and HeLa cells	reduces the Aβ production in HeLa cells	[306]
*Spatholobus suberectus Dunn*	ethanolic	in vitro and Aβ1-42	90.5% of protein aggregation inhibition	[297]
*Scutellaria pinnatidifa*	dichloromethane, n-butanol	in vitro, α-synuclein, PC12 cells, and dopaminergic neurons	dose-dependently inhibits protein fibrillization and protects against amyloid-induced cytotoxicity	[335]
*Thymus vulgaris*	ethyl acetate	In vitro, IAPP, and HeLa cells	attenuates IAPP aggregation and protects cells from amyloid-induced toxicity	[304]
*Uncaria* *rhynchophyla*	ethanolic, methanolic, aqueous	in vitro and Aβ	inhibits Aβ fibril formation and disassembles preformed Aβ fibrils	[336]
*Uncaria tomentosa*	Water	in vitro, Aβ, and tau	inhibits Aβ40 and tau protein amyloid fibril formation and disrupts preformed Aβ42 and tau protein fibrils to amorphous non-toxic aggregates	[337]
*Vigna angularis*	ethanolic	in vitro, Aβ1-42, and *drosophila models*	dose-dependently inhibits protein fibrillization and reduces the Aβ level in the brain of Aβ-overexpressing *Drosophila*	[338]
*Washingtonia filifera seed*	aqueous, methanolic, ethanolic	in vitro and IAPP	inhibits α-amylase, α-glucosidase enzyme activity, and IAPP fibril formation	[339]
*Withania somnifera*	aqueous	in vitro and Aβ1-42	reduces protein fibrillation in a concentration-dependent manner and inhibits cholesterol-induced Aβ1-42 aggregation	[340]
	residual material of chloroform-methanol extract	middle-aged and old APP/PS1 Alzheimer’s disease transgenic mice	reduces amyloid plaques, β-amyloid peptides levels, and oligomers in the brains of middle-aged and old APP/PS1 Alzheimer’s disease transgenic mice	[341]
	methanolic	*Caenorhabditis elegans* and BZ555 and NL5901 strains	exhibits neuroprotective and α-synuclein aggregation-mitigating effects	[342]
*Xysmalobium undulatum*	dichloromethane /methanol	in vitro and HeLa cells	reduces Aβ production in HeLa cells	[317]

## Data Availability

Data sharing is not applicable.
